# Synthesis, Characterization and Biological Investigations of Half-Sandwich Ruthenium(II) Complexes Containing Benzimidazole Moiety

**DOI:** 10.3390/molecules28010040

**Published:** 2022-12-21

**Authors:** Patrycja Rogala, Agnieszka Jabłońska-Wawrzycka, Grzegorz Czerwonka, Katarzyna Kazimierczuk, Katarzyna Gałczyńska, Sławomir Michałkiewicz, Justyna Kalinowska-Tłuścik, Marta Karpiel, Karel D. Klika

**Affiliations:** 1 Institute of Chemistry, Jan Kochanowski University of Kielce, 7 Uniwersytecka Str., 25-406 Kielce, Poland; 2 Faculty of Chemistry, Jagiellonian University, 2 Gronostajowa Str., 30-387 Kraków, Poland; 3 Institute of Biology, Jan Kochanowski University of Kielce, 7 Uniwersytecka Str., 25-406 Kielce, Poland; 4 Faculty of Chemistry, Gdańsk University of Technology, 11/12 G. Narutowicza Str., 80-233 Gdańsk, Poland; 5 Molecular Structure Analysis, NMR Spectroscopy Analysis Unit, German Cancer Research Center (DKFZ), Im Neuenheimer Feld 280, D-69120 Heidelberg, Germany

**Keywords:** ruthenium complexes, structural and spectroscopic studies, electrochemistry, X-ray diffraction, antibacterial and antibiofilm activity, molecular docking studies

## Abstract

Half-sandwich Ru(II) complexes belong to group of biologically active metallo-compounds with promising antimicrobial and anticancer activity. Herein, we report the synthesis and characterization of arene ruthenium complexes containing benzimidazole moiety, namely, [(η^6^-*p*-cymene)RuCl(bimCOO)] (**1**) and [(η^6^-*p*-cymene)RuCl_2_(bim)] (**2**) (where bimCOO = benzimidazole-2-carboxylate and bim = 1-*H*-benzimidazole). The compounds were characterized by ^1^H NMR, ^13^C NMR, IR, UV–vis and CV. Molecular structures of the complexes were determined by SC-XRD analysis, and the results indicated the presence of a *pseudo*-tetrahedral (piano stool) geometry. Interactions in the crystals of the Ru complexes using the Hirshfeld surface analysis were also examined. In addition, the biological studies of the complexes, such as antimicrobial assays (against planktonic and adherent microbes), cytotoxicity and lipophilicity, were performed. Antibacterial activity of the complexes was evaluated against *S. aureus*, *E. coli*, *P. aeruginosa* PAO1 and LES B58. Cytotoxic activity was tested against primary human fibroblasts and adenocarcinoma human alveolar basal epithelial cells. Obtained biological results show that the ruthenium compounds have bacteriostatic activity toward *Pseudomonas aeruginosa* PAO1 strain and are not toxic to normal cells. A molecular docking study was applied as a predictive source of information about the plausibility of examined structures binding with HSA as a transporting system.

## 1. Introduction

Nowadays, medicinal chemistry is facing a huge challenge, namely, drug resistance. The appearance of a reduced response of microorganisms to antimicrobial agents, as well as cancer cells’ response to applied chemotherapeutic drugs, is a serious problem in the treatment of different diseases. In drug discovery, there has been a positive trend in the design of new metal-complex-based drugs. Metal complexes exhibit unique chemical and biochemical properties that are absent in conventional bioorganic drugs [1,2]. Transition metals exhibit different oxidation states and can interact with a variety of molecules. These properties make transition metals promising pharmacological agents with unique therapeutic opportunities. However, their therapeutic applications are still an unexplored area of research. Ongoing research is expected to lead to the overcoming of some of the disadvantages associated with present drug therapies, including the reduction of side-effects, toxicity, widening the spectrum of activity and resistance. Moreover, it is predicted that further development of medicinal chemistry will go in the direction of establishing molecular activity mechanisms of metallodrugs in complex biological systems. Even though the most common metallodrugs have a few limitations and side effects, transition metal complexes are still the most widely used chemotherapeutic agents and make a large contribution to medicinal therapeutics [3]. Recently, researchers have been engaged in the discovery of the non-platinum complexes for anticancer therapy. The investigations of the mechanisms of some metal complexes action have demonstrated the broad variety of cell death pathways activated by them [4]. Bearing in mind that metal complexes can activate alternative pathways of cell death, possible opportunities for the treatment of tumors resistant to the currently available drugs have arisen. Copper and ruthenium complexes probably have greater potential over platinum complexes, showing reduced toxicity, a new mechanism of action, selectivity for tumor cells and the possibility of non-cross-resistance [4]. Similar to anticancer therapy, there is also an urgent need to look for new strategies to combat multidrug-resistant and infectious biofilms. Researchers are using various approaches to investigate new compounds with antimicrobial activity. So far, various inhibitors of different pathways and antimicrobial peptides [5,6,7], as well as organic and inorganic synthetic compounds [8,9,10,11,12] with antibacterial activity, have been identified and studied. After the success of transition metal coordination compounds as antibacterial and anticancer agents (Ag, Cu, Ru, Au, Zn, Mn) [13,14,15,16,17], it was found that the use of this group of compounds has enormous biomedical potential.

An interesting alternative are ruthenium complexes that are preferable because of their unique biochemical properties, such as the ability to form a labile complex, to have different oxidation states under physiological conditions and to mimic iron ions [18,19]. Among the different ruthenium compounds, arene ruthenium compounds belong to a well-established family of robust metal–organic molecules that have played an important role in the development of organometallic chemistry. They have attracted a lot of interest due to their promising anticancer and antibacterial activity, as well as catalytic potential in a wide range of organic reactions. One of the sources of arene ruthenium complexes is a dimer formulated as [(η^6^-arene)Ru(μ-Cl)Cl]_2_. Dimers have been found to undergo cleavage of the chloride bridges with various monodentate or bidentate ligands (L) to give the monomeric compounds [(η^6^-arene)RuCl_2_L] or [(η^6^-arene)RuClL]. The presence of the aromatic π-ligand as part of the ruthenium complex stabilizes and protects the metal center, preventing rapid oxidation to Ru(III). However, the arene moiety that is strongly coordinated to the ruthenium can be modified by simply attaching different substituents. The three other coordination sites opposite the arene ligand can be occupied by a wide variety of ligands with N-, O-, S- or P donor atoms, as well as halogen ions. The formed complexes can be neutral, mono- or dicationic and often result in metal–donor atom bonds useful for biological activity. Additionally, significant information can be obtained by investigating the effect of ligand structure on the properties of transition metal complexes. This is a good way to tune the biological properties of the arene ruthenium complexes. Research on arene–ruthenium(II) complexes, conducted in a wide range, has been involved, usually in terms of their anticancer activity. Studies on this type of complexes as antibiofilm agents are rather rare. However, considering the etiology of many pathological changes (e.g., bacterial and viral infections, neoplastic ulcers), it is important to test the antimicrobial activity of potential therapeutic agents. Epidemiological data indicate that wound infections are common in cancer patients. Wounds are the result of infiltrating neoplastic cells or may be a consequence of medical procedure complications [20,21,22]. Generally, cancer patients have an opportunistic infections. Therefore, the combination of biostatic/biocidal activity of the tested complexes and their anticancer action seems to be justified. The possibility of using complexes that simultaneously limit the development of cancer cells and protect against bacterial infections would be a great solution. Of course, it should be emphasized that the described properties do not always go hand in hand, which is confirmed by our previous research [23,24,25,26,27]. The antibiofilm effect depends on many factors, such as the structure of the complex—the type of central ion, ligand, the presence of chloride ions or its properties. It should also be noted that a selective effect on specific groups of microorganisms (depending on bacterial structure) is often observed.

Our project involves studies on the chelation complexes of ruthenium ions in different oxidation states with benzimidazole and pyridine derivatives (N,O– or N,N–donor ligands) in order to use them as antibiofilm and/or anticancer agents [23,24,25,26,27]. Our previous studies indicated that benzimidazole and pyridine derivatives were able to bind metal ions as N,O– or N,N–donors, forming stable five-membered chelate rings. In an N,N–donor ligand, the nitrogen-donating fragments (pyridine-like atoms) ligated to the ruthenium center and stabilized the +II or +III oxidation state of the metal [23,24]. Thereby, the presence of an N,N–donor ligand in [(η^6^-*p*-cymene)RuCl(pybim)] (pybim = 2-pyridin-2-yl-1*H*-benzimidazole) enhanced the antibiofilm and anticancer activity of the complex [24]. In turn, N,O–donor ligands formed stable complexes with Ru(III) or Ru(IV) ions [25]. However, their presence diminished the antibiofilm effect of complexes.

Thus, in this work, we attempt to investigate how the antibiofilm and anticancer activities will be affected by replacing the pyridyl substituent with a carboxylic one in arene Ru(II) complexes. In other words, we study the effect of the N,O–donor ligand on the activity of the Ru(II) arene complex. In addition, we also attempt to combine anticancer and antibiofilm properties in one compound, which would lead to the improvement of new and promising biological active agents.

In this work, we estimated the biological activity of two half-sandwich arene ruthenium complexes synthesized from carboxylic ligand containing benzimidazole moiety.

Herein, several aspects are investigated: (i) crystal structure determination and the physicochemical characterization of the obtained ruthenium complexes, (ii) the electrochemical behavior of Ru(II) complexes, (iii) the evaluation of antibacterial and antibiofilm activity of Ru compounds against selected bacteria strains and the (iv) the assessment of the antitumor activity of the examined complexes. To develop efficient anticancer agents, it is necessary to study the possibility of transport in biomembranes and interactions with serum proteins acting as drug carriers. Human serum albumin (HSA) is the most abundant circulating protein present in human blood plasma, which binds and transports endogenous and exogenous molecules. Therefore, HSA has been the protein of choice for many investigators to study drug protein interaction [28,29,30,31,32,33]. It is also known that HSA accumulates in tumor tissues, thus drugs binding to HSA are carried to diseased tissues. As a consequences, the toxic effect against normal cells is decreased [32,33]. A molecular docking study was used as a predictive source of valuable information about the plausibility of examined structures binding with HSA. The role of lipophilicity in determining the overall quality of drug candidate molecules is also of paramount importance. Therefore, such a study was also conducted.

## 2. Results

### 2.1. Preparation of the Complexes

**Scheme 1:**
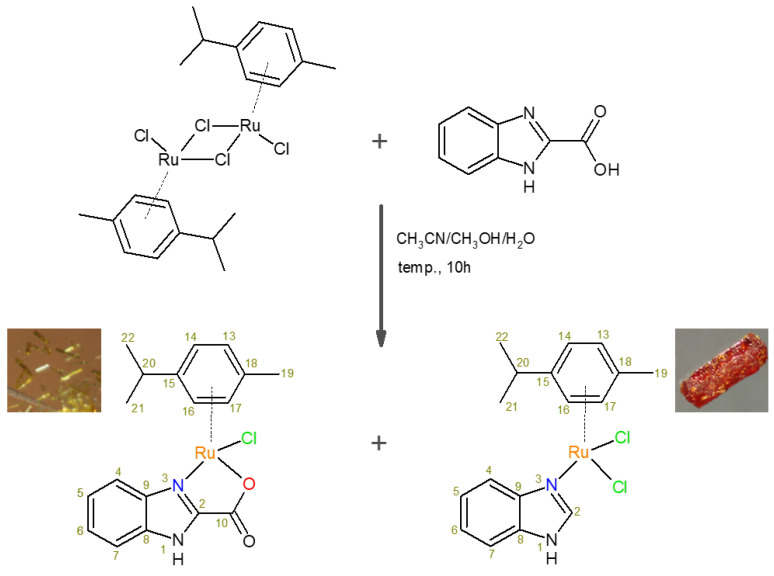
Synthetic route to the complexes [(η^6^-*p*-cymene)RuCl(bimCOO)] (**1**) and [(η^6^-*p*-cymene)RuCl_2_(bim)] (**2**).

**Scheme 2:**
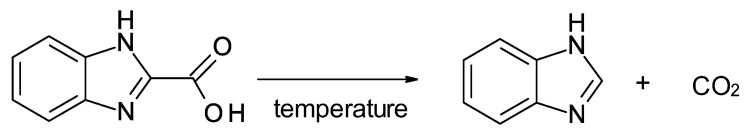
Decarboxylation process of 1*H*-benzimidazole-2-carboxylic acid.

The interaction between commercially available chloro-bridged dimeric arene ruthenium complex [(η^6^-*p*-cymene)Ru(µ-Cl)Cl]_2_ and 1*H*-benzimidazole-2-carboxylic acid in methanol/acetonitrile unexpectedly led to the formation of two neutral mononuclear ruthenium complexes—[(η^6^-*p*-cymene)RuCl(bimCOO)] (**1**) and [(η^6^-*p*-cymene)RuCl_2_(bim)] (**2**) (Scheme 1). The reaction conditions in which the ruthenium complex was synthesized with 1*H*-benzimidazole-2-carboxylic acid resulted in the decarboxylation process of the ligand. As is known, benzimidazoles containing a carboxyl group in the 2-positions readily undergo decarboxylation on heating [34]. A schematic reaction of the bimCOOH decarboxylation process is presented in Scheme 2.

The structural characterization of the obtained compounds was established by IR, ^1^H, ^13^C and ^15^N NMR spectroscopy, and the purity of the ruthenium complexes was confirmed by elemental analysis. The ruthenium complexes are air-stable and are soluble in common organic solvents like methyl, ethyl alcohols, acetonitrile, DMSO and dichloromethane.

### 2.2. Spectroscopic Characterization

Spectral data assignment (IR, NMR and UV–vis) for the isolated compounds provided important indications of their structures. The FT-IR spectrum of the 1*H*-benzimidazole-2-carboxylic acid revealed the existence of a zwitterionic state. The ligand has ionized COO^−^ and NH^+^ groups (Table S1). Based on the changes in the IR spectrum of complex **1** (compared to the uncoordinated ligand), the existence of the chelate N,O–donor coordination was established. Compound **1** exhibits the shifts in the ν_C=C_ and ν_C = N_ vibrations towards higher values of the wavenumbers (1530–1426 cm^−1^) in relation to the free ligand (1516–1424 cm^−1^), which proves the coordination of the nitrogen atom of bimCOOH to the metal ion. The coordination of the oxygen atom is evidenced by the characteristic strong bands at 1640 cm^−1^ and 1329 cm^−1^, corresponding to the asymmetric and symmetric stretching vibrations of the carboxylate. The Δν value calculated according to the spectroscopic criterion given by Deacon and Phillips (Δν = [ν(COO^−^)_asym_ − ν(COO_−_)_sym_]) [35] indicates the monodentate coordination of the carboxylate group in the complex **1** (Δ = 311). The selected IR assignments are given in Table S1. Analysis of the IR spectrum of compound **2** revealed the absence of the band corresponding to the ν_COO_^−^ group. However, the ν_C=C_ and ν_C=N_ stretches in the region of 1541–1415 cm^−1^ are observed. Slight changes in the shift of these vibrations, when compared with the frequencies of the free ligand, suggest that a nitrogen atom is involved in coordination with the central ion.

The structures of the arene ruthenium complexes were established also by NMR spectroscopy. The spectra of the ligand and the ruthenium(II) complexes were consistent with the proposed structures, and the assignments of the ^1^H, ^13^C and ^15^N nuclei were accomplished by the standard application of 1D and 2D correlation spectra, confirmed in some cases with molecular modeling calculations [36] using standard methodology for the calculation of chemical shifts [37]. The NMR study revealed that bimCOOH was surprisingly unstable in DMSO and readily decarboxylated to yield bim. The ligand sample was otherwise free of impurities. In contrast, the complex **1** was stable in DMSO, and the benzimidazole moiety did not decarboxylate, and, thus, clearly, complexation impedes decarboxylation by tying up the imine-type sp^2^-hybridized nitrogen that must be involved in the decarboxylation reaction pathway. The expected signals of coordinated *p*-cymene and bimCOOH or bim were observed in the proton spectra for compounds **1** and **2**. In the ^1^H NMR spectra of the complexes, a doublet as well as septet for protons of the isopropyl moiety, a singlet for the methyl group in the arene and two doublets for the protons of the *p*-cymene ring were observed. In addition, ^1^H NMR revealed singlet signals at 14.01 ppm (complex **1**) and 13.42 ppm (complex **2**), which is assigned to the resonance of NH protons on the benzimidazole ring. The resonances of the protons of the aromatic ring of the ligand were shifted upfield with respect to those of the uncoordinated ligand, which confirms the coordination of the ligand to the ruthenium(II) ion. The ^13^C NMR spectra also show signals expected for the metal-coordinated arene group and the benzimidazole moiety. Both complexes display the signals between 77 and 103 ppm that correspond to the aromatic carbons of the *p*-cymene. The carbon atoms of the ligand’s benzimidazole ring can be found in the regions 113.71–145.53 ppm for **1** and 115.02–146.32 ppm for **2**. Their significant resonance shifts (especially C2 and C9; (Δδ_coord._)), compared to the free ligand, indicate the coordination of the ligand with the central ion (Table S2). Moreover, the ^13^C NMR spectrum of complex **1** contains one singlet belonging to the COO^–^ group at 163.72 ppm, which is strongly shifted to higher frequencies with respect to the corresponding starting material (Δδ = +4.99 ppm). This effect is related to the coordination of the carboxyl group with the electropositive metal ion. The mode of coordination of the ligand in a solution was also confirmed in the ruthenium(II) complexes from the ^15^N NMR data. It was found that the resonance signals corresponding to nitrogen atoms in the benzimidazole moiety were shifted after coordination with the metal ion (Table S2).

The electronic spectra (in methanolic solution) of the Ru(II) complexes and 1*H*-benzimidazole-2-carboxylic acid are shown in Figure S1. The UV–vis spectral data for the compounds tested are given in Table 1.

Comparative analysis of the UV–vis spectra of the ligand and the ruthenium complexes indicated that strong bands below 300 nm are related to the intraligand (IL) electronic transitions—π→π*/n→π*. The absorption bands found in the range 326 to 411 nm are associated with metal-to-ligand charge transfer (MLCT) transitions. These transitions arise from the excitation of electrons from the metal t_2g_ level to the unfilled molecular orbitals π* level of the ligands. The lowest energy absorption bands (~410 nm) were also assigned as MLCT based on the high values of the molar extinction coefficient (ε = 262–407 dm^3^/mol∙cm) (Table 1). The expected *d*–*d* transition bands are difficult to identify in the spectra of the ruthenium(II) complexes due to their low intensity. Additionally, absorption *d*–*d* bands of Ru(II) compounds are masked by the stronger charge transfer bands. In the low-spin complexes discussed, the electronic *d*–*d* transitions are forbidden by both the Laporte and spin selection rules. This makes the *d*–*d* transitions very difficult to detect when they occur in the same region as the CT bands [38].

### 2.3. Description of the Molecular and Crystal Structure of Complexes **1** and **2**

**Figure 1:**
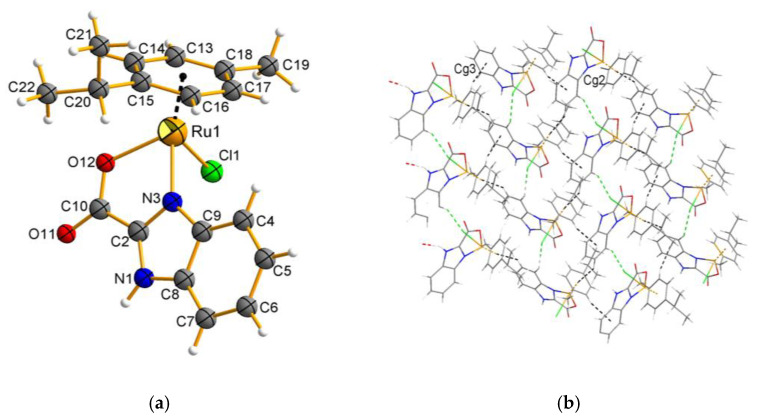
Molecular structure of complex **1** (**a**). Crystal packing with marked supramolecular interactions of C–H⋅⋅⋅Cl and C–H⋅⋅⋅π types forming *zig-zag* chains (view along *z-axis*) (**b**).

**Figure 2:**
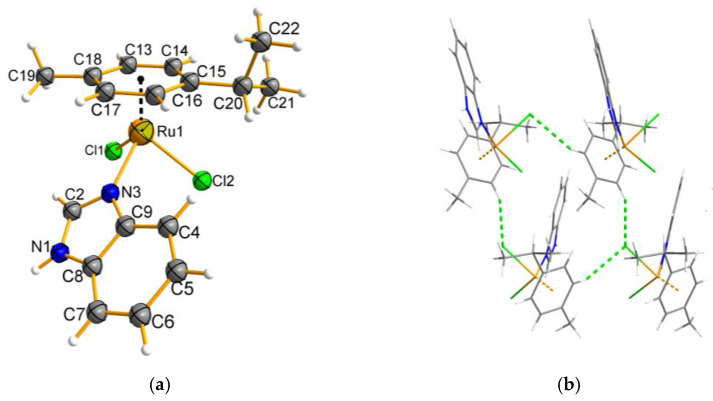
Molecular structure of complex **2** (**a**). Crystal packing with marked supramolecular interactions of C–H···Cl types constructing tetramer (view along *c* axis) (**b**).

Single crystals of complexes **1** and **2**, suitable for structure determination, were obtained by the slow evaporation of their CH_3_CN/CH_3_OH/H_2_O solutions at room temperature, which crystallized in the *P*2_1_/n and *P*na2_1_ space groups, respectively (Table S3). The molecular diagrams of complexes **1** and **2** are shown in Figure 1 and Figure 2, while Table 2 and Table 3 give a comparison of important bond distances and angles.

Complex **1**, as a yellow plate, crystallizes with one neutral molecule in each asymmetric unit. The molecular structure of complex **1** shows the metal center being coordinated by the N,O–donor ligand and a chloride, which occupy the positions like the legs of the piano stool, while the arene ring occupies the remaining coordination site like the seat of the piano stool (Figure 1a). As a result of deprotonation, bimCOOH changes from the carboxyl to the carboxylate form. The bidentate action of the ligand allows the formation of a stable five-membered chelate ring with ruthenium(II) ions. The ligand (bimCOOH) plane is situated perpendicular to the *p*-cymene, presumably to reduce the steric interaction with each other. The C–C bond distances (benzene ring of benzimidazole) become more inequivalent, and two short C4–C5 and C6–C7 (~1.389 Å) and two long (C5–C6, C8–C9) bonds (~1.406 Å) and the rest in between C4–C9 and C7–C8 (~1.395 Å) indicate the extensive electron delocalization along the ligand backbone. This is in agreement with observations of our previous work [24]. The geometry around ruthenium can be described as distorted *pseudo*-tetrahedral. The *p*-cymene is 1.6583(6) Å away from the ruthenium ion. The Ru–N and Ru–O bonds in complex **1** are slightly asymmetric, and their distances range from 2.0876(2) to 2.1163(1) Å. They are comparable to those reported for related compounds [39,40]. The Ru–Cl bond is definitely longer (2.4015(5) Å). The values of the valence angles range from 76.68(2) to 134.97(4)° and, as can be seen, significantly differ from those predicted for a regular polyhedron. Another description of coordination environment is known as the *pseudo*-octahedral three-legged piano stool structure.

The analysis of packing in the complex **1** network indicated the presence of intermolecular interactions of the N–H⋅⋅⋅O, C–H⋅⋅⋅Cl and C–H⋅⋅⋅π types. The classical H-bond is strong, with H⋅⋅⋅A and D⋅⋅⋅A distances of 2.6795(2) and 1.8622(2) Å, respectively. Structural units are linked via C–H⋅⋅⋅π interaction into *zig-zag* chains (Figure 1b). The C–H group of the benzimidazole ring and arene participate in this interaction. The chains are held together via C–H⋅⋅⋅π and C–H⋅⋅⋅Cl interactions. The interactions involve the *p*-cymene/benzimidazole hydrogen atoms (C13-H13/C4-H4) and the benzimidazole ring or chloride ion (Figure 1b, Table 4).

Figure 2a for complex **2** shows that the Ru(II) ion is coordinated with one monodentate ligand (bim), one arene and two chloride ions giving rise to a {RuNC_3_Cl_2_} chromophore. The similar structure of the arene complex was reported by Matveeevskaya [41] but with a different synthesis procedure and X-ray measurement conditions. The structural analysis confirmed the presence of bim that was generated by the decarboxylation of benzimidazole-2-carboxylic acid. The geometry of the ruthenium coordination environment can be visualized as distorted *pseudo*-tetrahedral or as the *pseudo*-octahedral three-legged piano stool structure. The N-donor of benzimidazole and two chloride ions are in the legs positions of the piano stool, while the arene ring occupies the remaining coordination site as the seat of the piano stool. The bond distance of Ru–N is short, with a value of 2.112(2) Å. This parameter is similar to that found in other arene Ru(II) complexes having a benzimidazole ligand [41,42]. Moreover, the benzimidazole C–C bond distances become more inequivalent, with C4–C5 and C6–C7 (~1.375 Å) shorter than the other one C5–C6 (1.413 Å) and the other three C–C bonds (C4–C9, C8–C9, C7–C8) that are in between (~1.399 Å) suggesting electron delocalization in the ligand. The *p*-cymene group is connected to the Ru(II) center by distances ranging from 2.154(2) to 2.203(2) Å through η^6^ bonding. The longest Ru–Cl bonds reach a value of 2.4185(6) and 2.4297(6) Å. The distances are also longer than that observed for complex **1**.

Interactions of the N–H⋅⋅⋅Cl and C–H⋅⋅⋅Cl types contribute to the stability of the [(η^6^-*p*-cymene)RuCl_2_(bim)] crystal lattice. The classical hydrogen bonds of the N–H⋅⋅⋅Cl type belong to three-centered interactions (bifurcated donor hydrogen bonds), and they are between the N1 atom of benzimidazole and two chloride ions of the adjacent molecule. An interesting element of this architecture are the C–H⋅⋅⋅Cl interactions that were used in the construction of hydrogen tetramers (Figure 2b). It is worth emphasizing that we noticed the presence of rare trifurcated acceptor interactions of the C–H⋅⋅⋅Cl type in the network (Table 4).

### 2.4. Hirshfeld Surface Analysis

**Figure 3:**
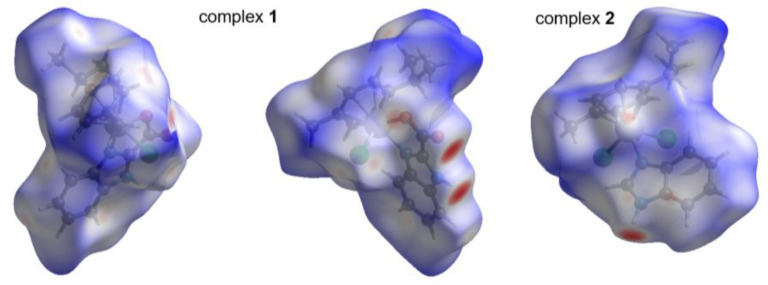
The Hirschfeld surfaces highlight the relevant *d*_norm_ surface patches associated with the specific contacts for arene Ru(II) complexes.

**Figure 4:**
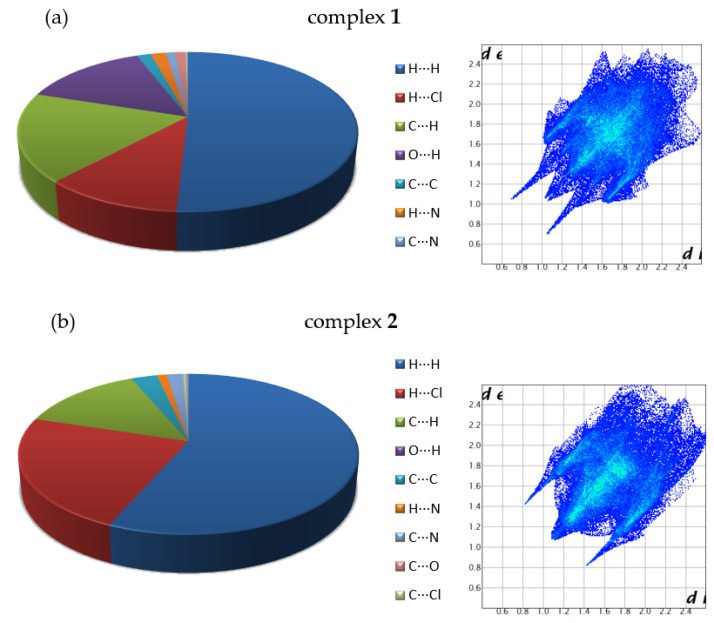
The 2D fingerprint plots of all the intermolecular interactions for complexes **1** (**a**) and **2** (**b**) with percentage of interaction.

The Hirshfeld surface method is extremely useful for revealing intermolecular interactions, and its popularity is constantly growing [43]. As a reliable procedure, it predicts a molecular stacking pattern based on structural data and crystal engineering. Investigating intermolecular interactions is crucial to understanding not only the molecular stacking structures but also some properties of crystals. It is worth noting, for example, the wide range of the applications of this method in describing intermolecular interactions including hydrogen bonding, π-stacking and lone pair−π stacking. The molecular Hirshfeld surface of the Ru(II) complexes are presented in Figure 3. Three-dimensional (3D) Hirshfeld surface maps were obtained using red–white–blue *d*_norm_ surface maps (surface resolution −0.0667 to 1.656 Å), where red indicates shorter contacts with negative *d*_norm_ values, white indicates close van der Waals contacts with zero *d*_norm_ values, and blue indicates longer contacts with positive *d*_norm_ values. A shape index provided a measure of the “shapes” of molecules in crystal lattices, enabled complementarity between molecules to be identified and provided C–H–π interaction information. The overall 2D fingerprint plots obtained by the Hirshfeld surface analysis shown in Figure 4, were also studied. The main reciprocal intermolecular interactions (H⋅⋅⋅H, H⋅⋅⋅Cl, C⋅⋅⋅H, O⋅⋅⋅H) were obtained using the 2D fingerprint plot and the 3D *d*_norm_ surfaces of the arene Ru(II) complexes.

Definitely, H⋅⋅⋅H contacts have a largest share in complex 1, the contribution of which to the overall surface contacts was 50.8% (Figure 4). The C⋅⋅⋅H/H⋅⋅⋅C contacts with a 18% proportion appeared as a pair of characteristic wings in the 2D fingerprint plot. The presence of a pair of characteristic wings in the fingerprint plot decomposed into O⋅⋅⋅H/H⋅⋅⋅O contacts, contributing 14.2% to the HS. The H⋅⋅⋅Cl/Cl⋅⋅⋅H contacts (Figure 4) contributed 11.1% to the HS and formed a pair of spikes. The other contacts accounted for a total of 4.6% to the overall surface. For complex 2, the H⋅⋅⋅H contacts were dominant and constituted 56.6% contribution to the Hirshfeld surface. The H⋅⋅⋅Cl/Cl⋅⋅⋅H interactions contributed second-most (23.2%) to the total Hirshfeld surface. Another significant spot in the Hirshfeld surfaces corresponded to reciprocal C⋅⋅⋅H intermolecular contacts, the contribution of which was 13.8%. The C⋅⋅⋅C, C⋅⋅⋅Cl, C⋅⋅⋅N and H⋅⋅⋅N contacts were interpreted as minor and had a share of 1.1 to 2.9%.

### 2.5. Electrochemical Behavior of Ruthenium Complexes

**Figure 5:**
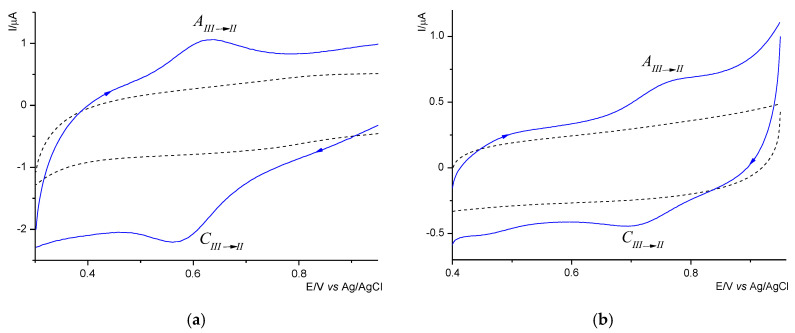
Cyclic voltammograms of complex **1** (**a**) and complex **2** (**b**) (each at a concentration of 1 mM) recorded in acetonitrile–ethanol mixture containing 0.1 M TBAPF_6_. Lines (–) and (---) represent the curves of the ruthenium complexes and the ligand/supporting electrolyte, respectively (CV conditions: GCE, Ø = 2 mm, T = 25 °C).

Cyclic voltammetry (CV) is a powerful and popular electrochemical technique that can be used for analyzing drugs and other antimicrobial agents. We employed CV to study qualitative information about electron transfer processes under various conditions, such as the diffusive or adsorptive nature of electrode process, its reversibility and the formal redox potential. The CV curves were recorded from the initial potential 0.3 vs. Ag/AgCl (1M NaCl) with scan rates of 50, 100 and 200 mV/s on glassy carbon electrodes. To verify the reversibility of the redox couples and the number of electrons exchanged, CV diagnostic criteria, Δ*E*_p_ = *E*_pa_ − *E*_pc_ (*E*_pa_, *E*_pc_ denote the potentials of the anodic and cathodic peaks, respectively), were applied [44,45]. It should be noted that the ligand appears to not be electroactive in the experimental conditions. The voltammetric curves of arene ruthenium complexes are illustrated in Figure 5. Table 5 lists the voltammetric data of the studied compounds.

The voltammograms of Ru(II) complexes show only one redox pair. In both CV curves of complexes, the anodic signal appeared (Figure 5) as a poorly shaped peak, as well as the cathodic peak. As we established, the redox pair corresponds to the oxidation/reduction process of Ru(II)↔Ru(III). For complex **1**, we observed that, as the scan rate increases, the value of the peak-to-peak separation also increases significantly (Table 5). This feature indicates the irreversible nature of the electrode process. Moreover, the calculated Δ*E*_p_ values are well above the theoretical value of 0.058 V, especially at higher scan rates (Table 5). The applied criterion, *E*_p_ − *E*_p/2_ = 0.047.7V/(nα) (where α is the transfer coefficient, the value of which is close to 0.5) [45] made it possible to specify that one electron is exchanged in the process. It was found that the dependence of the peak current, *I*_pa_, on the square root of the scan rate is non-linear. This discrepancy with the Randles–Sevčik equation proves the adsorption-controlled redox process. It is also confirmed by the linear dependence of log *I*_pa_ on log v, for which the slope of 0.93 is a value close to the theoretical one (1.0) [45]. In turn, in complex **2**, the peak-to-peak separation for the Ru(II)/Ru(III) redox pair does not change with the scan rate. The constant potential of the anodic and cathodic peaks (Table 5), for which the Δ*E*_p_ values of 0.06 V are similar to the theoretically expected, indicates a reversible character of the electrode process. The diagnostic criterion used, *E*_pa_ − *E*_pc_ = 0.058 V/n, indicates that one electron is transferred. For this electrochemical process, we noticed that, according to the Randles–Sevčik equation, peak current *I*_pa_ increases linearly with the square root of the scan rate v, which indicates the diffusion-controlled redox process. Moreover, the diffusive nature of the process is also confirmed by the linear dependence of log *I*_pa_ on log v, for which the slope of 0.56 is a value close to the theoretical one (0.5) [45].

### 2.6. Antimicrobial Assays

The bacteriostatic activities of the compounds (arene Ru complexes, Ru(II) precursor and ligand) against representative Gram+ and Gram− bacteria were evaluated by determining their minimum inhibitory concentration (MIC), using a broth microdilution assay. The MIC parameter is considered a valuable tool to verify the in vitro activity of new antimicrobial agents. The antibacterial efficacy of the complexes examined was compared with a commonly commercial drug, streptomycin. The results are summarized in Table 6.

When all of the strains were incubated in the presence of the ruthenium(II) precursor, no relevant effects against either control strains were observed. In turn, fairly moderate effectiveness was exhibited by bimCOOH, which acted at the highest concentration tested towards *P. aeruginosa* PAO1. As shown in Table 6, the ruthenium complexes had different activity profiles across the strains. The Ru(II) complexes were effective against Gram-negative rods, generally. Complex **1** inhibited the growth of two of the four bacteria at a concentration of 1 mM (432 μg/mL). These results refer to the opportunistic *P. aeruginosa* species. Meanwhile, complex **2** exhibited inhibitory action against *P. aeruginosa* PAO1 and *E. coli*. This is an interesting observation, taking into account that the *Pseudomonas aeruginosa* species exhibit great phenotypic and genetic diversity. In other cases, the complexes turned out to be ineffective against bacteria at the highest tested concentrations. On this basis, the antibacterial activity of the ruthenium complexes can be assessed as rather weak to moderate.

**Figure 6:**
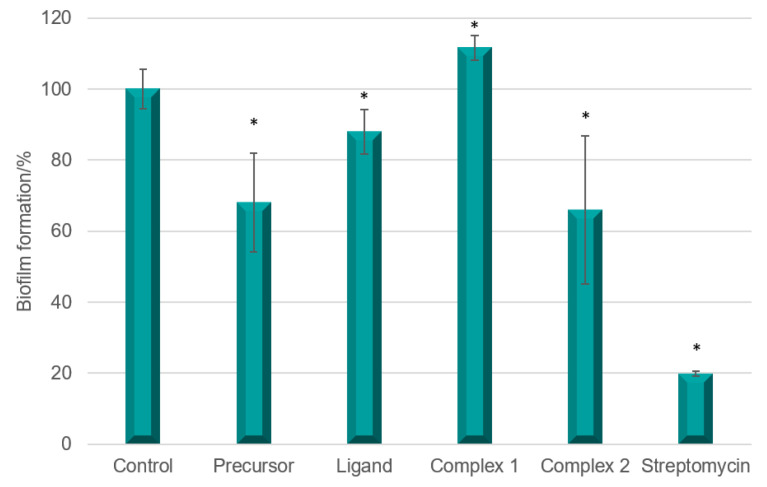
*P. aeruginosa* PAO1 biofilm formation in the presence of Ru(II) precursor, free bimCOOH and arene Ru complexes (concentration of compounds—1 mM). The absorbance of the control was considered to represent 100% of biofilm formation (results were considered significant when compared to control; * *p* < 0.05. Data are presented as mean ± SD, *n* = 4).

To determine the efficacy of potential antimicrobial agents to prevent the formation of biofilms, a crystal violet assay is frequently used. Crystal violet staining is a simple test performed to detect the maintained adherence of cells, and it provides quantitative information about the relative density of cells adhering to the material. The effect of the ruthenium complexes on biofilm formation by laboratory *P. aeruginosa* strain was analyzed, in terms of total biomass and cellular metabolic activity, by epifluorescence microscopy. The results of the biological activity of the compounds against the *P. aeruginosa* PAO1 are presented in Figure 6.

As the results indicate, the free bimCOOH exhibited rather weak effectiveness (23% of inhibition) at the tested concentration and was more active than its ruthenium complex (Figure 6). Moreover, it seemed complex **1** promoted biofilm formation. It is worth mentioning that the system we studied previously with the same ligand and Ru(III) ion showed an activity of 17% [25]. In comparison, compound **2** was able to inhibit biofilm formation, reducing biomass by 34% when the concentration was 1 mM.

**Figure 7:**
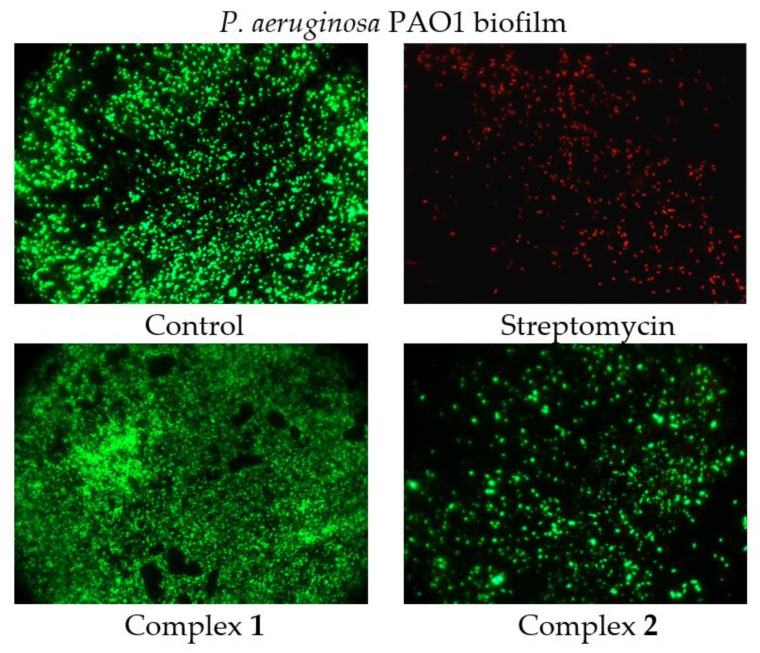
Epifluorescence microscopy images of *P. aeruginosa* PAO1 biofilm treated with 1 mM of arene Ru(II) complexes. Biofilm was stained with nucleic acid stains using the FilmTracer™ LIVE/DEAD Biofilm Viability Kit (live cells are represented by the color green; dead cells are represented by the color red). The epifluorescence microscopy images were captured at 1000× magnification.

To investigate the cell viability and morphological changes of the *P. aeruginosa* PAO1 biofilm treated with ruthenium complexes, a LIVE/DEAD biofilm viability kit (Invitrogen) was used, and fluorescence microscope (EFM) observations were performed. The LIVE/DEAD assay staining solution is a mixture of two fluorescent dyes (SYTO-9 and propidium iodide) that differentially label live and dead cells. The live cell dye, SYTO-9, penetrates bacterial membranes freely and fluoresces when bound to DNA, staining intact, viable cells green. Propidium iodide enters only the cells with compromised membranes and stains the dead cells, generating red fluorescence. Figure 7 shows the effect of the ruthenium complexes on the *P. aeruginosa* PAO1 biofilm architecture. As can be seen in the representative images, the cells are well spread and viable on the control surface. Contrary to the surfaces treated with complex **2**, the arene Ru(II) compound with bim led to the morphological and structural damage of the *P. aeruginosa* PAO1 biofilm and caused the aggregation of cells, although, to a small extent, it led to the death of bacterial cells, which we observed as red areas. For complex **1**, the image shows the area with live cells evenly distributed, showing no characteristic aggregates.

### 2.7. Cytotoxicity Activity

To evaluate the cytotoxicity of the ruthenium complexes, a colorimetric MTS assay was carried out against primary human fibroblasts (VH10) and adenocarcinoma human alveolar basal epithelial cells (A549). In addition, a quantitative assessment of apoptosis was performed by flow cytometry based on Annexin-V FITC and propidium iodide (PI) double staining. The results obtained in both the MTS assay and flow cytometry showed that the tested ruthenium complexes in the concentration range of 30 to 1000 µM are neither cytotoxic to A549 lung cancer cells nor to normal VH10 skin cells (IC_50_ above 1000 µM) (Figure S2, Table S4). Two other research teams also studied the cytotoxic activity of the ruthenium–benzimidazole complex [41,46]. The Ru complex, synthesized by scientists from Russia, was evaluated against human breast adenocarcinoma (MCF-7) and human hepatocellular carcinoma (HepG2) cell lines [41]. The half maximal inhibitory concentration (IC_50_) of this compound on MCF-7 was 47.3 ± 0.8 μM (concentration of 25 μM). In turn, the HepG2 cell count decreased by more than half at 50 μM concentration. Vock and coworkers conducted a cytotoxicity experiment on mouse TS/A tumor cells and nontumorigenic human breast (HBL-100) cells. The IC_50_ values for TS/A and HBL-100 were 573 μM and 601 μM, respectively [46]. We suspect that the our ruthenium complexes do not show cytotoxic activity because they diffuse poorly across cell membranes.

### 2.8. Solution Stability and Lipophilicity of Complexes

The stability of a compound in solution and lipophilicity are important properties considered in drug molecule design [47]. The stability of the synthesized ruthenium complexes was assessed using UV–vis spectroscopy in a methanolic solution and in an aqueous solution with the addition of dimethyl sulfoxide (49/1 *v*/*v*). The experiments were performed by monitoring the solutions at different time intervals up to 24 h to mimic the biological test conditions. No significant changes in the UV–vis spectra over time were noticed, signifying that the complexes are stable in the tested solvents. The UV–vis spectra of the complexes in H_2_O/DMSO were also recorded after 48 h. In the case of complex **2**, a slight band shift near to 330 nm was observed. The time-dependent electronic absorption spectra of the complexes are depicted in Figures S3 and S4.

The lipophilic properties of compounds can help to explain the distribution of the drug candidates and predict their transport in different biological systems. In our experiment, the lipophilicity of the ruthenium complexes was estimated by the n-octanol/water partition coefficients (log P), which were determined using the shake-flask method. The calculated log *P* values for compounds **1** and **2** were 0.525 and 0.497, respectively. According to the literature [48], substances with log *p* values in the range from −2 to 0 are polar and accumulate mainly in the nuclei and lysosomes, while compounds with log *p* > 0 are lipophilic and accumulate preferentially in mitochondria and the endoplasmic reticulum. Our results indicate that the studied compounds possess a rather hydrophobic character and probably accumulate in the mitochondria and endoplasmic reticulum, but this statement needs further experiments.

### 2.9. Binding Ability of the Studied Ru(II) Complexes with HSA

**Figure 8:**
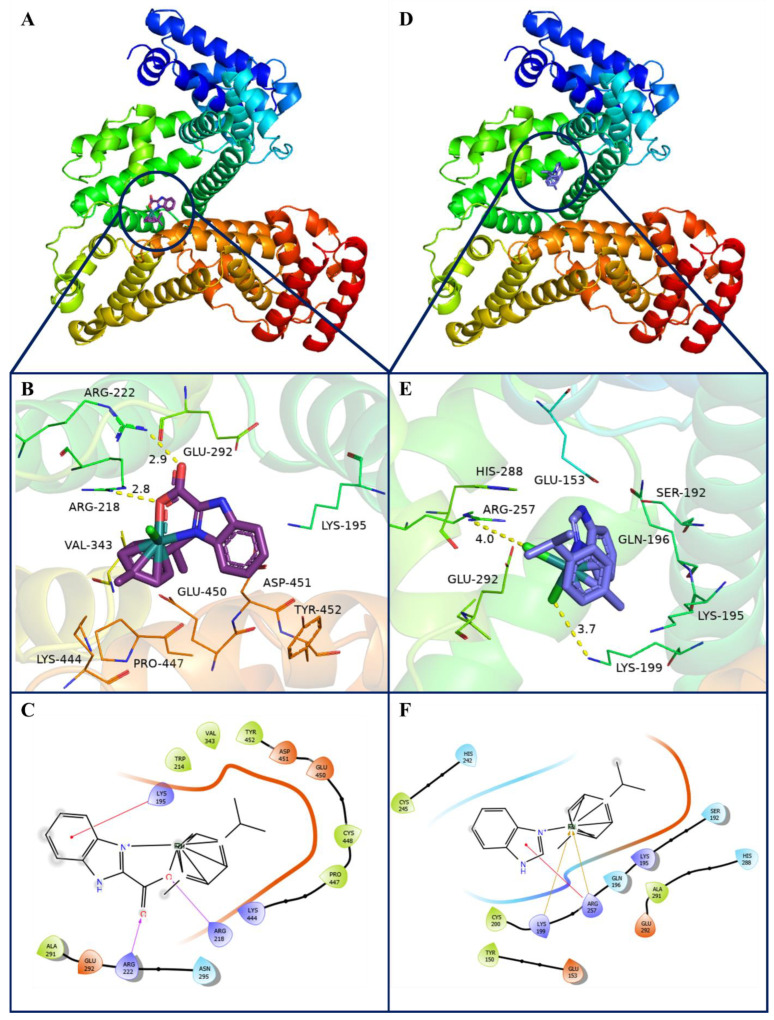
The best-scored poses obtained by molecular docking experiments for HSA with Ru(II) complex **1** (**A**) and Ru(II) complex **2** (**D**). HSA residues involved in forming interactions with compounds **1** and **2** are shown in panels (**B**,**E**), respectively. Furthermore, 2D ligand–protein interactions plots were generated for HSA with compound **1**’s (**C**) and compound **2**’s (**F**) best poses.

Human serum albumin (HSA) is the main binding protein for metallodrugs present in blood plasma, and its ability to bind metal-based drugs is well studied. HSA belongs to the group of serum proteins that can act as a drug carrier of anticancer agents; hence, it is essential to study the binding of ruthenium complexes with this key protein [49]. Two primary drug-binding sites of HSA are well established. They differ in size and binding specificity and are called site I and site II. The main properties of site I are a large size and flexibility. This binding site is located around Trp214, which is an important amino acid capable of forming π–π interactions with ligands. Other characteristics are four basic residues, Lys195, Lys199, Arg218 and Arg222, located at the entrance to binding site I [50]. The interactions of synthesized complexes with HSA were predicted using the molecular docking method. Simulations were performed for compounds **1** and **2** with HSA binding site I, and the best results for those structures, with a scoring function only slightly worse compared to warfarin re-docking results, are presented in Figure 8. The GOLDscore results for the best-ranked poses were 66.60 for warfarin, 55.84 for compound **1** and 42.67 for compound **2**. The crucial protein–ligand interactions stabilizing the HSA-compound **1** complex are hydrogen bonds. Atoms involved in the mentioned interactions are oxygen atoms of the carboxyl group of Ru(II) complex **1,** which is located in the vicinity of Arg218 and Arg222, with the shortest distances between interacting atoms being 2.8 Å and 2.9 Å, respectively. Additionally, a few weaker interactions are stabilizing complex **1** within the HSA binding site I, e.g., weak hydrogen bonds with Glu450; hydrophobic contacts with Val343, Lys444 and Pro447; and interactions involving π-electrons with Lys195, Asp451 and Tyr452 (Figure 8B). In the case of the HSA-compound **2** adduct, the important stabilizing interactions are formed between two chlorine ligands of the Ru(II) complex with Lys199 and Arg257, for which distances are 3.5 Å and 4.0 Å, respectively. The additionally formed interactions with residues constituting binding site I are shown in Figure 8D. Among those weak hydrogen bonds with His288 and Glu153 can be distinguished, as well as hydrophobic contacts with the side chains of Lys195 and Glu292, or the aromatic ring interacting with Gln196. Furthermore, 2D protein–ligand interaction maps of the best poses for each compound were generated and are presented in Figure 8C and 8F for **1** and **2**, respectively. A molecular docking study can be applied as a predictive source of valuable information about the plausibility of examined structures binding with HSA. Data obtained for compounds **1** and **2** suggest that both investigated compounds are capable of interacting with HSA at a level comparable to warfarin and, potentially, may be distributed by this protein in the human circulatory system.

## 3. Discussion

As indicated by biological studies, tested ruthenium(II) complexes are characterized by low antibiofilm and anticancer activity. Comparing the antibiofilm activity of complex **1** to the previously studied Ru(II) chelate complex with a benzimidazole derivative containing a pyridyl substituent (pybim = 2-pyridin-2-yl-1*H*-benzimidazole) [(η^6^-*p*-cymene)RuCl(pybim)] (78% inhibition of *P. aeruginosa* PAO1 biofilm formation), a significant decrease in activity can be seen [27]. Both complexes contained a ruthenium ion in the +II oxidation state, *p*-cymene, a chloride ion and a benzimidazole derivative ligand with a substituent in the 2-position (-py or -COOH). Pybim as ligand, used in our previous papers [24,27], belongs to the group of N,N–donor ligands, and the one studied in this paper belongs to the group of N,O–donor ligands. It seems that the presence of ligands from the last group generates complexes with lower antibiofilm activity [25]. Although the carboxyl group and the pyridine nitrogen atom give a stable system with the central ion (bond lengths indicate a high affinity of the donor atoms to the Ru(II) ion) resulting from the formation of a five-membered chelate ring. As is known [51], the presence of the -COOH group in the ligand affects the electron density distribution in the benzimidazole moiety (pulling the electron density away from the ring) and impacts on the electron-donor property of the ligand. It is important to note that the increase in electron density at the metal center has consequences in the weak reducing properties of the compound. This may also be the reason for the weak antibiofilm activity of the complex presented in this work. On the other hand, although the pyridyl substituent in the complex [(η^6^-*p*-cymene)RuCl(pybim)] pulls the electron density away from the ring, it shows weaker electron donor properties. This causes the significant antibiofilm activity increase. It should also be noted that the complex of Ru(III) with bimCOOH [25] showed a low antibiofilm activity of 17%. Based on our previous studies, we can postulate that good activity (64% inhibition of biofilm formation) was exhibited by the Ru(IV) complex with a pyridine derivative substituted with as many as two carboxyl groups [25]. In the case of complex **2**, wherein the ligand (bim) is a monodentate, a system containing two chloride ions is formed. The premises indicate that the analogous literature complexes of Ru(II) and Ru(III) show good and very good antimicrobial activity [52,53]. Therefore, the moderate antibiofilm activity of complex **2**, representing only 34% of the reduction in biofilm formation, is puzzling. It should also be considered whether the conducted anticancer studies reflect the real effect of the complexes on cell lines and do not reveal the significant role of the solvent used in the test and the composition of the medium for cell lines. As was investigated, the presence of a mixture of H_2_O/DMSO (2%) or methanol did not affect the stability of complexes **1** and **2** (24 h), but the use of DMSO may lead to their destabilization. In addition, our previous studies [54] showed that ruthenium complexes have a high affinity for amino acids and proteins (BSA, K_SV_ = 5.2∙10^4^ M^−1^). In this paper, the plausible binding of Ru(II) complexes to HSA, the drug carrier of anticancer agents present in blood plasma, was studied by the molecular docking method. The capability of the interacting of the studied complexes with HSA at a level comparable to warfarin suggests a possible transporting system of the investigated compounds in the human circulatory system. However, this can be a problem in the assay, because compounds with peptide groups will effectively compete with the proper target—eukaryotic cells—and thus cause too weak of a therapeutic effect. On the other hand, it is known that carboxyl substituents in ligands may affect the weakening of bioactive agent permeation across the biomembrane. It can be also concluded that the ruthenium complexes are hy-drophobic in nature as lipophilicity parameters indicated.

## 4. Materials and Methods

1*H*-benzimidazole-2-carboxylic acid and (*p*-cymene)ruthenium(II) chloride dimer were purchased from Sigma Aldrich and used as received. Analytical-grade solvents (acetonitrile, methanol) were purchased from Chempur.

### 4.1. Chemical Experiments

#### 4.1.1. Syntheses of [(η^6^-p-cymene)RuCl(bimCOO)] (**1**) and [(η^6^-p-cymene)RuCl_2_(bim)] (**2**)

Complexes (**1**) and (**2**) were synthesized by the reaction of [(η^6^-*p*-cymene)Ru(µ-Cl)Cl]_2_ (0.1531 g; 0.25 mmol) with 1*H*-benzimidazole-2-carboxylic acid (bimCOOH) (0.0811 g; 0.5 mmol) in a 20 mL mixed solution (acetonitrile: methanol; 1V: 1V) and a few drops of water. The mixture was heated (ca. 65 °C) at reflux and stirred for 10 h. Afterwards, the reaction solution was left to crystallize slowly at room temperature. After a few days, yellow crystals of complex **1** suitable for X-ray investigation was obtained. Complex **1** was filtered off and dried in a vacuum box. The product was collected with 88% yield, with a melting point of 288 to 289 °C.

Anal. Calc. (%) for RuC_18_H_19_N_2_ClO_2_: C, 50.05; H, 4.44; N, 6.49; Found: C, 49.84; H, 4.37; N, 6.44; FT-IR (cm^−1^): 3070 (m), 3039 (w), 2960 (ms), 2906 (ms), 1640 (vs), 1590 (s), 1530 (s), 1492 (s), 1477 (vs), 1426 (ms), 1391 (vs), 1329 (vs), 1308 (s), 1160 (m), 1148 (m), 1059 (ms), 1028 (vs), 870 (vs), 834 (vs), 747 (vs), 650 (s), 604 (vs), 528 (s). ^1^H NMR (400 MHz, DMSO-*d*_6_, *δ* ppm): 1.09, 1.14 (6H, d, (C_21/22_**H**_3_)_2_C_20_H, *J*_H16_ = 6.9 Hz); 2.17 (3H, s, C_19_**H**_3_); 2.71 (1H, m, (C_21/22_H_3_)_2_C_20_**H**, *J*_H21,H22_ = 6.9 Hz); 5.81–6.06 (4H, C_13_**H**, C_14_**H**, C_16_**H**, C_17_**H** (*p*-cymene), *J*_H13,H14_ = 6.0 Hz, *J*_H16,H17_ = 5.9 Hz); 7.49–8.07 (4H, C_4_**H**, C_5_**H**, C_6_**H**, C_7_**H** (ligand)); 14.01 (1H, s, N–H); ^13^C NMR (100 MHz, DMSO-*d_6_*, *δ* ppm): 18.34 (**C_19_**H_3_); 21.72, 21.78 (**C_21/22_**H_3_)_2_C_20_H); 30.69 (C_21/22_H_3_)_2_**C_20_**H); 77.30–100.70 (**C_13_**–**C_17_** (*p*-cymene)); 113.71–145.53 (**C_2_**–**C_9_** (benzimidazole ring)); 163.72 (**C_10_**); ^14^N NMR (50 MHz, *δ* ppm): −228.41 (N3); μ_eff_ = 0 μ_B_ for a low-spin Ru^2+^ (electron configuration of _44_Ru^2+^ [Kr] 4d^6^).

The orange filtrate was left. The slow evaporation of the filtrate afforded a very small quantity of small red crystals (complex **2**, 9% yield), one of which was employed for X-ray structure determination. Melting point: 231–232 °C. Anal. Calc. (%) for RuC_17_H_20_N_2_Cl_2_: C, 48.11; H, 4.76; N, 6.60; Found: C, 47.69; H, 4.71; N, 6.59; FT-IR (cm^−1^): 3140 (ms), 3105 (m), 3034 (w), 2970 (ms), 2922 (ms), 1541 (ms), 1489 (s), 1473 (ms), 1415 (s), 1384 (ms), 1305 (ms), 1246 (s), 1145 (w), 1058 (ms), 1009 (ms), 891 (ms), 872 (s), 743 (vs), 669 (s), 616 (s), 552 (s), 522 (s). ^1^H NMR (400 MHz, DMSO-*d*_6_, *δ* ppm): 1.18, 1.21 (6H, d, (C_21/22_**H**_3_)_2_C_20_H *J*_H16_ = 6.9 Hz); 2.09 (3H, s, C_19_**H**_3_); 2.84 (1H, m, (C_21/22_H_3_)_2_C_20_**H**, *J*_H21,H22_ = 6.9 Hz); 5.63–6.05 (4H, C_13_**H**, C_14_**H**, C_16_**H**, C_17_**H** (*p*-cymene), *J*_H13,H14_ = 6.1 Hz, *J*_H16,H17_ = 5.8 Hz); 7.27–7.76 (4H, C_4_**H**, C_5_**H**, C_6_**H**, C_7_**H** (ligand)); 8.38 (1H, s, C_2_**H**) 13.42 (1H, s, N–H); ^13^C NMR (100 MHz, DMSO-*d_6_*, *δ* ppm): 18.42 (**C_19_**H_3_); 22.12, 22.19 (**C_21/22_**H_3_)_2_C_20_H); 30.71 (C_21/22_H_3_)_2_**C_20_**H); 79.83–102.71 (**C_13_**–**C_17_** (*p*-cymene)); 115.02–146.32 (**C_2_**–**C_9_** (benzimidazole ring)); ^14^N NMR (50 MHz, *δ* ppm): −219.76 (N3); μ_eff_ = 0 μ_B_ for a low-spin Ru^2+^ (electron configuration of _44_Ru^2+^ [Kr] 4d^6^).

#### 4.1.2. Physical Measurements

The percentage compositions of the elements (C, H and N) of the synthesized complexes were determined using a Vario Micro Cube Elemental Analyser CHNS. The IR spectra were recorded on a Nicolet 380 FT-IR type spectrophotometer in the spectral range 4000 to 500 cm^−1^ using the ATR-diffusive reflection method. NMR spectra were acquired using a Bruker Avance III instrument equipped with a 5 mm, normal configuration probe with z-axis gradient capability at a field strength of 9.4 T operating at 400.26, 100.64 and 40.56 MHz for ^1^H, ^13^C and ^15^N nuclei, respectively, in *d*_6_-DMSO at 25 °C. Pulse widths were calibrated following the described protocol [55]. The chemical shifts of ^1^H and ^13^C nuclei are reported relative to internal TMS (δ_1H,TMS_ = 0 ppm and δ_13C,TMS_ = 0 ppm), while the chemical shifts of ^15^N nuclei are reported relative to external 90% CH_3_NO_2_ in CD_3_NO_2_ (δ_15N,CH3NO2_ = 0 ppm). General NMR experimental and acquisition details for 1D ^1^H, ^13^C, ^13^C-DEPT, ^15^N-INEPT, and selective ^1^H{^1^H}-NOESY and standard, gradient-selected 2D ^1^H{^1^H}-COSY, ^1^H{^13^C/^15^N}-HSQC, ^1^H{^13^C/^15^N}-HMBC, and f1-decoupled C, H correlation have been previously described [56,57,58,59] for routine spectral assignment and structural analysis. Furthermore, 2D spectra were generally acquired using NUS. The molecular modeling of ligands using Gaussian 03 [36] was conducted at the RB3LYP/TZVP level of theory for geometry optimization and at the RB3LYP/CC-PVTZ level of theory for the calculation of chemical shifts that were subsequently calibrated using the results from a set of small molecules with known chemical shifts as per standard methodology [37]. UV–vis measurements in methanolic solutions were performed on a V-630 UV–vis spectrophotometer from Jasco using 1 cm cuvettes against methanol as reference solutions. All absorbance measurements were recorded at room temperature and the concentrations were 3.09 × 10^−5^ M and 3.14 × 10^−5^ M for ruthenium complexes and 4.10 × 10^−5^ M for the ligand. Magnetic measurement was carried out with a magnetic susceptibility balance (Sherwood Scientific) at room temperature by Gouy’s method, using Hg[Co(NCS)_4_] as a calibrant. The voltammetric experiments were conducted on a Model M161E electrochemical analyzer cooperating with the EALab 2.1 software (mtm-anko, Cracow, Poland). In a conventional three-electrode cell, a glassy carbon electrode (GCE) with a 2 mm diameter, A = 0.0314 cm^2^ (Mineral, Warsaw, Poland), and a platinum wire were used as a working electrode and a counter (auxiliary) electrode, respectively. The potential was measured against the external silver/silver chloride reference electrode (Ag/AgCl) with 1 M NaCl solution (Mineral, Warsaw, Poland). To avoid water leakage, the reference electrode was isolated from the solution by a salt bridge with a frit of Victor Glass. Pure argon was used for deoxygenating the solutions prior to voltammetric investigations. The electrochemical studies of the ruthenium(II) complexes and free ligand were carried out in a mixture of CH_3_CN/EtOH (3:2, *v/v*; Chempur, analytical-grade) with 0.1 M tetrabutylammonium hexafluorophosphate (TBAPF_6_) (Fluka, electrochemical grade) as a supporting electrolyte. The concentration of the ligand and the Ru(II) complexes for the cyclic voltammetry measurements was 1 × 10^−3^ M. The measurements were performed at room temperature (25 ± 1 °C).

#### 4.1.3. Crystal Structure Setermination and Refinement (SC-XRD)

For the arene ruthenium(II) complexes, single-crystal X-ray data were collected on a STOE IPDS 2T diffractometer, equipped with a STOE 6.67 IP detector. A X-ray MoKα radiation source with a graphite monochromator was used. The determination of the unit cell and diffraction data collection were carried out at 120 K in a stream of dry nitrogen (Oxford CryoSystem, Long Hanborough OX29, UK). The data collection, data reduction, absorption correction and refinement were performed using the X-Area 1.75 (STOE and Cie GmbH, Darmstadt, Germany, 2015) software package [60]. The structures were solved by direct methods, and all non-hydrogen atoms were refined with anisotropic thermal parameters by the full-matrix least squares procedure based on F^2^. Final refinements were carried out using the SHELX system [61] run under the control of the WinGX program [62]. WinGX was also used to prepare the final version of the CIF files. Hydrogen atoms were refined in a geometrically idealized position with isotropic temperature factors 1.2 times the equivalent isotropic temperature factors Ueq of their attached atoms (1.5 for CH_3_ groups). The crystallographic data and some details of the structural refinement are summarized in Table S5. The figures were made using DIAMOND [63] software. CCDC 2223311 and 2223312 contain the supplementary crystallographic data for **1** and **2**, respectively. These data can be obtained free of charge from the Cambridge Crystallographic Data Center via https://www.ccdc.cam.ac.uk/structures/ (accessed on 30 November 2022).

#### 4.1.4. Hirshfeld Surface Analysis

Molecular Hirshfeld surface calculations were performed using the Crystal Explorer package *ver.* 3.1 [64]. When the .cif file of the title compounds was entered into the Crystal Explorer program, all of the bond lengths to hydrogen were automatically modified to the standard neutron values (CH = 1.083 A). Hirshfeld surface analysis included the descriptor *d*_norm_ and the *shape index* [43]. The calculations and details of analysis were made as described in [25]. The molecular Hirshfeld surfaces of complexes **1** and **2** were generated using a standard (high) surface resolution with the 3D *d*_norm_ surfaces mapped over a fixed color scale of −0.0667 (for **1**)/−0.432 (for **2**) (red) to 1.656 (for **1**)/1.453 (for **2**) Å (blue). The *shape index* was mapped in the color range of −1 to 1 (for **1** and **2**). The color encodes the normalized distance to the nearest nuclei and thus conveniently illustrates the “strength” of all types of the intermolecular contacts present. In turn, the fingerprint plots provide a quantitative measure of the intermolecular interactions on the surface.

### 4.2. Biological Tests

#### 4.2.1. Antibacterial Activity—Minimum Inhibitory Concentration

The in vitro antibacterial activity of the free ligand and the ruthenium(II) complexes was investigated by the broth microdilution method in order to determine the minimum inhibitory concentration (MIC). The bacteria used in this study were *Staphylococcus aureus* ATCC 6538P, *Escherichia coli* ATCC 8739 *Pseudomonas aeruginosa* PAO1 and *Pseudomonas aeruginosa* LES B58 (clinical isolate). The *E. coli* ATCC 8739 and *S. aureus* 6538P were selected for minimum inhibitory concentration tests due to their original use as model strains for antimicrobial testing. The *P. aeruginosa* PAO1 and LES B58 were used because of their multidrug resistance. The *P. aeruginosa* PAO1 and LES B58 isolates were derived from the international *Pseudomonas aeruginosa* reference panel [65].

The bacteria were cultivated in TBS (Trypticasein soy broth) medium (Biocorp, Warsaw, Poland) for 18 h at 37 °C with shaking (160 rpm). Overnight bacterial cultures were diluted with fresh TSB medium at a ratio of 1:100. The suspensions were placed in the 96 wells of microtiter plates and supplemented with solutions of the ruthenium complexes or the ligand at concentrations of 1 mM, 0.5 mM, 0.25 mM, 0.125 mM and 0.0625 mM. After that, the plates were incubated overnight at 37 °C. Control tubes were maintained for each test batch. These included: negative control (bacterial culture in the medium without growth inhibitory agent) and positive control (antibiotic control—streptomycin). Compounds for testing were stored as 2 mM stock solutions in aqueous solutions with dimethyl sulfoxide. The final concentration of DMSO did not exceed 2% by volume, and an equivalent volume of DMSO with no compound was added to the control cultures. It is to be mentioned that DMSO did not show any traceable antimicrobial activity at the studied concentrations, and thus the solvent did not influence the biological activity of the tested compounds. The MIC parameter was recorded as the lowest concentration of the test substance that had no visible turbidity (no bacterial growth) when compared with the control, using the Infinite M200 PRO microplate reader (Tecan, Mannedorf, Switzerland). All experiments were conducted in triplicate.

#### 4.2.2. Inhibition of *P. aeruginosa* PAO1 Biofilm Formation

*Pseudomonas aeruginosa* PAO1, a model strain for biofilm formation testing, was selected to investigate the biofilm inhibition process. Crystal violet staining was used to evaluate the inhibition effect of the ruthenium complexes on biofilms of *P. aeruginosa* PAO1. Briefly, a bacterial culture for biofilm measurements was incubated overnight at 37 °C as described in the previous paragraph. The diluted cell suspension of *P. aeruginosa* PAO1 was placed into the wells of a 96-well microtiter plate containing the test compounds in a concentration range of 0.0625 mM to 1 mM (the ruthenium complexes were prepared by dissolving compounds in distilled water with the addition of dimethyl sulfoxide (2%), which was previously tested for antibacterial activity and found to have no antibacterial activity. The starting stock solution was of 2 mM concentration). The inoculated plates were incubated at 37 °C for 24 h. The negative control (bacterial culture in the medium) and positive control (antibiotic control—streptomycin) were used as references. After 24 h, planktonic cells were removed, and the wells washed thrice with sterile water. The biofilm was stained by adding 0.01% crystal violet solution to wells for 15 min. Then, the plates were washed thrice with water to remove unbound crystal violet. The elution of the dye from the biofilm was achieved by adding 80% ethanol into the wells and incubating for 15 min at room temperature. To estimate total biofilm biomass, the optic density of the resulting solution was measured at 595 nm on an Infinite M200 PRO microplate reader. Tests were performed in three independent experiments. The measurement results, expressed in absorbance units, were converted into percentages to allow the comparison of numerical data obtained in different experiments.

#### 4.2.3. Live/Dead Staining of the Bacterial Biofilm

The cell viability and morphological changes of *P. aeruginosa PAO1* biofilm after the ruthenium complexes treatment was analyzed using fluorescence microscopy. Initially, the *P. aeruginosa* PAO1 cells were cultivated in 6-well microtiter plates on glass coverslips in TSB broth at 37 °C for 24 h without shaking. Then the culture was supplemented with solutions of the study compounds (concentration: 1 mM). After 24 h incubation at 37 °C, the coverslips were carefully washed with sterile water to remove non-adherent cells. Afterwards, microcolonies formed on the glass surface were stained with a FilmTracer™ LIVE/DEAD^®^ Biofilm Viability Kit (Invitrogen, Carlsbad, CA, USA) in accordance with the manufacturer’s protocol. Images were collected with an Axio Scope.A1 epifluorescence microscope (Carl Zeiss, Jena, Germany). The experiments were repeated three times to obtain consistent results.

#### 4.2.4. Cytotoxicity Activity (MTS Assay and Apoptosis Test by Flow Cytometry)

Primary human fibroblasts (VH10) were cultured at 37 °C in a humidified 5% CO_2_ atmosphere in plastic dishes in Dulbecco’s modified eagle medium (DMEM) supplemented with 10% heat-inactivated fetal bovine serum, 2 mM of l-glutamine and antibiotics (100 units/mL penicillin and 100 µg/mL streptomycin). Adenocarcinoma human alveolar basal epithelial A549 cells (ATCC^®^ CCL-185™) were provided by American Tissue Cell Collection. A549 cells were cultured in F-12K medium (Sigma Aldrich Chemicals, Rockville, MD, USA) supplemented with 10% fetal bovine serum (Invitrogen, Carlsbad, CA, USA), 2 mM L-glutamine (Sigma Aldrich Chemicals, USA) and antibiotics (100 units/mL penicillin and 100 μg/mL streptomycin (Invitrogen, CA, USA)) at 37 °C. Both cell lines were cultured in a humidified 5% CO_2_ atmosphere.

Cytotoxic properties of the ruthenium(II) complexes were measured by a MTS Cell Proliferation Assay Kit (Abcam, ab197010) in accordance with the manufacturer’s instructions. Cells were seeded into a 96-well plate and incubated with the ruthenium complexes in the concentration range of 30 to 1000 µM for 24 h at 37 °C in a humidified atmosphere of 5% CO_2_. After incubation, a solution of MTS (3-(4,5-dimethylthiazol-2-yl)-5-(3-carboxymethoxyphenyl)-2-(4-sulfophenyl)-2*H*-tetrazolium) was added to each well and incubated at 37 °C for 4 h. The measurement of the absorbance of the solution related to the number of live cells was conducted on a TECAN Spark Microplate Reader (TECAN, Männedorf, Switzerland) at 490 nm. All samples were tested in three independent experiments. Results were normalized to the control. Inhibitory concentrations (IC_50_) represent the concentrations of the tested samples required to inhibit 50% cell proliferation and were calculated from the mean values of the data from the wells.

The Annexin V-FITC apoptosis detection test was used to confirm the MTS test. The analysis of apoptotic and necrotic cell death was carried out in three independent experiments according to the manufacturer’s instructions using a Annexin V-FITC apoptosis detection Kit I (BD Pharmingen, Franklin Lakes, NJ, USA) and Becton Dickinson LSR II flow cytometer. Briefly, cells were suspended in 100 µL of the binding buffer and incubated with 5 µL of annexin V-FITC and 5 µL of propidium iodide (PI) at room temperature for 20 min in the dark. The fluorescence was determined using a Becton Dickinson LSR II flow cytometer. A computer system (CellQuest Pro, Becton Dickinson) was used for data acquisition and analysis. Data for 20,000 events were stored. A cell gate containing A549 or VH10 cells was established on the basis of forwarding and side light scatter. Four different populations of cells were detected with the Annexin V-FITC kit: normal cells that were Annexin-negative and PI-negative and that expressed no fluorescence, early apoptotic cells that were Annexin-positive and PI-negative and that expressed green fluorescence, late apoptotic/necrotic cells that were Annexin-positive and PI-positive and that expressed green and orange fluorescence, and necrotic cells that were Annexin-negative and PI-positive and that expressed orange fluorescence.

#### 4.2.5. Determination of Stability and Lipophilicity

The solution stability of the ruthenium(II) complexes was studied by UV–vis spectroscopy in a methanolic solution and in an aqueous solution with the addition of dimethyl sulfoxide (the final concentration of DMSO did not exceed 2% by volume—conditions as in biological studies). The concentrations of the prepared solutions of the ruthenium complexes were 1 × 10^−4^ M. The UV–vis spectra were recorded on a Jasco 630 UV–vis spectrophotometer, after the dissolution of the investigated compounds, after 2 h, after 24 h as well as after 48 h, over the wavelength range of 200 to 600 nm.

The lipophilicity of the synthesized complexes was determined by the shake-flask method [66]. Solutions of the investigated compounds were prepared at concentration of 3 × 10^−4^ M in the DMSO+water mixture. Then, an equal volume of n-octanol was added to the ruthenium-containing aqueous solutions, and the mixtures were shaken mechanically for 72 h at room temperature. At the equilibrium condition, the n-octanol layer was separated carefully from the water layer for the metal compound analysis. The concentration of the complex in each separated phase was evaluated using UV–vis spectroscopy and used for the calculation partition coefficient (log P) values, according to the following equation: log P = log([complex]_octanol phase_/[complex]_water phase_). For each compound, three measurements were performed (the values of the log P are the mean ± standard deviation).

#### 4.2.6. Molecular Docking Study

The crystal structure of HSA (human serum albumin) in complex with warfarin bound to the binding site I (PDB ID 1H9Z [67]) was downloaded from Protein Data Bank (PDB) [68]. The initial geometries of the studied Ru^2+^ complexes were taken from the final .cif files. The protein and ligand structures’ preparation and generation of 2D interaction maps were conducted using the Maestro 12.7 program [69]. Molecular docking experiments were performed using GOLD 2021.3.0 (Genetic Optimisation for Ligand Docking) software [70]. The region of binding site I was defined in a way to include all of its residues within the docking sphere, centered at Trp214 (with a radius of 15 Å). The number of genetic algorithm runs was set to 20 for each compound. The GOLDscore scoring function [71] was used in this study to assess the obtained poses. Warfarin was re-docked as a reference ligand. Data obtained from molecular docking were analyzed and visualized using PyMOL [72].

#### 4.2.7. Statistical Analysis

Statistical analysis was performed using one-way analysis of variance (ANOVA). Significance was set at *p* < 0.05.

## 5. Conclusions

We have developed a procedure for the synthesis of Ru(II) complexes with a benzimidazole derivative, which allows us to obtain two arene compounds of this metal with the following formulas: [(η^6^-*p*-cymene)RuCl(bimCOO)] and [(η^6^-*p*-cymene)RuCl_2_(bim)]. The complexes were characterized by crystal structure, spectroscopic and electrochemical techniques. In both compounds, the geometry around the Ru(II) ion is *pseudo*-tetrahedral. Packing analysis pointed out that the intermolecular classical hydrogen bonds and rare weak interactions (such as trifurcated C–H∙∙∙Cl interaction) significantly contribute to structure stabilization and played a decisive role in the supramolecular architecture and the application of the examined complexes. Moreover, the spectroscopic data presented correlate well with the structural parameters observed. Electrochemical results pointed out that the ruthenium ions are in a +II oxidation state and that the complexes exhibit reducing properties. Such valuable properties can be useful related to a mechanism of action in biological structures. Ruthenium is considered an iron homologue, which suggests its affinity to cancer cells. It is very important for transport in a biomembrane to combine lipophilicity and hydrophilicity in one compound. In turn, transport in biological media coupling with molecules with an important biological function could allow targeted chemotherapy. Although the cytotoxicity parameters for ruthenium complexes, related to the A549 line, are not the best, molecular docking studies suggests that both examined Ru(II) complexes are capable of interacting with HSA and, potentially, may be distributed by this protein in the human circulatory system. The binding was confirmed using molecular docking studies that revealed the binding site of Ru complexes and the type of bonding, as well as the residues and functional groups involved in the binding. Arene Ru(II) complexes bind exclusively to site I of HSA through weak hydrogen bonds, hydrophobic contacts or aromatic ring interaction which are the primary forces stabilizing the protein–ligand complex. Biological studies show that the antibiofilm activity of complex **2** toward *Pseudomonas aeruginosa* PAO1 strain is rather medium but better than for complex **1**. Moreover, the EFM microphotography reveled morphological and structural changes of the *P. aeruginosa* PAO1 biofilm, which were the result of the formation of cell aggregates.

## Figures and Tables

**Table 1:** UV–vis spectroscopic data for the ligand and the arene ruthenium(II) complexes.

Compound	Transition λ, nm (ε, dm^3^/mol∙cm)	
	π→π*/n→π*	MLCT dπ(Ru)→π*(L)/*d*–*d*
bimCOOH	234 (6936), 259 (7159), 291 (6665)	
Complex **1**	227 (8064), 293 (7153)	332 (1275), ~410 (405)
Complex **2**	213 (6554), 267 (3159), 273 (3119), 278 (2328)	326 (495), ~405 (260)

**Table 2:** Selected bond lengths (Å) and angles (°) for complex **1**.

Bond lengths (Å)			
Ru1-O12	2.1163(1)	Ru1-Cl1	2.4015(5)
Ru1-N3	2.0876(2)	Ru1-Ct1	1.6583(6)
**Valence angles (°)**			
O12-Ru1-N3	76.68(2)	Ct1-Ru1-Cl1	128.43(1)
O12-Ru1-Cl1	84.49(4)	Ct1-Ru1-N3	134.97(4)
N3-Ru1-Cl1	83.49(5)	Ct1-Ru1-O12	129.74(4)

Ct1 denotes centroid of C13-C14-C15-C16-C17-C18 ring (*p*-cymene).

**Table 3:** Selected bond lengths (Å) and angles (°) for complex **2**.

Bond lengths (Å)			
Ru1-N3	2.1120(2)	Ru1-Cl2	2.4297(6)
Ru1-Cl1	2.4185(6)	Ru1-Ct1	1.6574(3)
**Valence angles (°)**			
N3-Ru1-Cl1	86.83(6)	Ct1-Ru1-N3	128.25(6)
N3-Ru1-Cl2	83.76(6)	Ct1-Ru1-Cl1	125.50(2)
Cl1-Ru1-Cl2	86.95(2)	Ct1-Ru1-Cl2	130.72(2)

Ct1 denotes centroid of C13-C14-C15-C16-C17-C18 ring (*p*-cymene).

**Table 4:** Geometry of classical hydrogen bonds and supramolecular interactions of C–H⋅⋅⋅Cl and C–H⋅⋅⋅π types for complexes **1** and **2**.

Complex	D-H⋅⋅⋅A	D-H (Å)	H⋅⋅⋅A (Å)	D···A (Å)	∠D-H⋅⋅⋅A (°)
**1**	N1-H1⋅⋅⋅O11 _(2−x,1−y,−z)_	0.8796(2)	1.8622(2)	2.6795(2)	153.75(1)
	C4-H4⋅⋅⋅Cl1 _(−1+x,y,z)_	0.9498(2)	2.8195(5)	3.6588(2)	147.88(1)
	C21-H21C⋅⋅⋅Cl1_(1.5−x,0.5+y,0.5−z)_	0.9795(3)	2.805(5)	3.7293(3)	157.60(2)
	C5-H5⋅⋅⋅Cg2	0.9505(2)	2.6858(1)	3.5023(2)	144.36(2)
	C13-H13⋅⋅⋅Cg3	0.9502(2)	2.5874(1)	3.505(2)	162.43(2)
**2**	N1-H1⋅⋅⋅Cl1 _(x+0.5,−y+0.5,z)_	0.88	1.91	3.387(2)	115.90
	N1-H1⋅⋅⋅Cl2 _(x+0.5,−y+0,5,z)_	0.88	2.37	3.224(2)	163.80
	C2-H2⋅⋅⋅Cl1	0.9498(3)	2.7816(6)	3.2087(3)	108.27(4)
	C21-H21A⋅⋅⋅Cl(2)	0.9799(3)	2.2726(6)	3.4858(3)	134.73(2)
	C17-H17⋅⋅⋅Cl(2) _(x+1,y,z)_	0.9502(3)	2.8013(6)	3.5254(3)	133.71(2)
	C13-H13⋅⋅⋅Cl(2) _(−x+1,−y+1, z)_	0.9501(3)	2.7195(7)	3.5293(3)	143.57(2)

Cg2 denotes center of gravity of C13–C14–C15–C16–C17–C18 ring (*p*-cymene); Cg3 denotes center of gravity of C4–C5–C6–C7–C8–C9 ring (benzimidazole).

**Table 5:** Electrochemical data [in V vs. Ag/AgCl] of the Ru(II) arene complexes obtained by CV.

Complex	Scan Rate V·s^−1^	Ru(II)↔Ru(III)			
		*E*_pa_/V	*E*_pc_/V	Δ*E*_p_/V	*E*_1/2_/V
**1**	0.05	~0.635	~0.565	~0.07	~0.60
	0.10	~0.640	~0.560	~0.08	~0.60
	0.20	~0.645	~0.555	~0.09	~0.60
**2**	0.05	~0.780	~0.720	~0.06	~0.75
	0.10	~0.780	~0.720	~0.06	~0.75
	0.20	~0.790	~0.730	~0.06	~0.76

Δ*E*_p_ = |*E*_pa_ − *E*_pc_|, *E*_1/2_ = ½(*E*_pc_ + *E*_pa_).

**Table 6:** Results of the minimum inhibitory concentration (MIC) for Ru compounds evaluated, expressed in mM and μg/mL.

Compound	BACTERIA							
	*S. aureus* ATCC 6538P		*E. coli* ATCC 8739		*P. aeruginosa* PAO1		*P. aeruginosa* LES B58	
	mM	μg/mL	mM	μg/mL	mM	μg/mL	mM	μg/mL
Ru(II) precursor	>1	>612	>1	>612	>1	>612	>1	>612
bimCOOH	>1	>162	>1	>162	1	162	>1	>162
**1**	>1	>432	>1	>432	1	432	1	432
**2**	>1	>424	1	424	1	424	>1	>424
Streptomycin	0.0625	36	0.125	73	0.0625	36	0.5	291

## Data Availability

Data sharing not applicable.

## Supplementary Materials

The following supporting information can be downloaded at: https://www.mdpi.com/article/10.3390/molecules28010040/s1, Figure S1: UV–vis spectra of 1*H*-benzimidazole-2-carboxylic acid, complex **1** and complex **2** in methanol at 298 K.; Figure S2: The MTS percentage of VH-10 and A549 cells metabolic activity under an increasing dose of ruthenium complexes or their ligand alone after 24 h treatment.; Figure S3: UV–vis spectra of the complexes **1** (a) and **2** (b) in methanol solution, after preparation and after 24 h.; Figure S4: Time dependence of UV–vis spectra of the complexes **1** (a) and **2** (b) in H_2_O/DMSO mixture.; Table S1: The characteristic IR absorption frequencies (cm^–1^) of the ligand and the ruthenium(II) complexes.; Table S2: ^1^H, ^13^C and ^15^N NMR chemical shifts of ruthenium complexes in DMSO-*d*_6_. The coordination shifts (Δ_coord._) are shown in parentheses.; Table S3: Crystallographic data and structure refinement details for the arene Ru(II) complexes.; Table S4: Percentage of early and late apoptotic and necrotic A549 and VH-10 cells following treatment with ruthenium complexes measured by flow cytometry; mean of three independent experiments ± SD. IP—propidium iodide.

## Sample Availability

Samples of compounds **1** and **2** are available from the authors.
