# Supplementary material for: Synthesis, Characterization and Biological Investigations of Half-Sandwich Ruthenium(II) Complexes Containing Benzimidazole Moiety
Source: Molecules. 2022 Dec 21;28(1):40. doi: 10.3390/molecules28010040 (PMC9821818; doi:10.3390/molecules28010040)
Supplement: Supplementary file 1 [file molecules-28-00040-s001.zip › molecules-2104856-supplementary.pdf]

Electronic Supplementary Information

Synthesis, Characterization and Biological Investigations of Half-Sandwich Ruthenium(II) Complexes Containing Benzimidazole Moiety

Patrycja Rogala[1*], Agnieszka Jabłońska-Wawrzycka[1,2*], Grzegorz Czerwonka[3], Katarzyna Kazimierczuk[4], Katarzyna Gałczyńska[3], Sławomir Michałkiewicz[1], Justyna Kalinowska-Tłuścik[2], Marta Karpiel[2], Karel D. Klika[5]

[1]*Institute of Chemistry, Jan Kochanowski University of Kielce, 7 Uniwersytecka Str., 25-406 Kielce, Poland*
[2]*Faculty of Chemistry, Jagiellonian University, 2 Gronostajowa Str., 30-387 Kraków, Poland*
[3]*Institute of Biology, Jan Kochanowski University of Kielce, 7 Uniwersytecka Str., 25-406 Kielce, Poland*
[4]*Department of Inorganic Chemistry, Faculty of Chemistry, Gdańsk University of Technology, 11/12 G. Narutowicza Str., 80-233 Gdańsk, Poland*
[5]*Molecular Structure Analysis, NMR Spectroscopy Analysis Unit, German Cancer Research Center (DKFZ), Im Neuenheimer Feld 280, D-69120 Heidelberg, Germany*
Correspondence: patrycja.rogala@ujk.edu.pl; agnieszka.jablonska-wawrzycka@ujk.edu.pl

CONTENTS

1. Supporting tables……………………………………………….......S2 – S4

2. Supporting figures……………………………………………......S5 – S7

1. Supporting tables

Table S1. The characteristic IR absorption frequencies (cm^{-1}) of the ligand and the ruthenium(II) complexes.

Assignment	bimCOOH	Complex 1	Complex 2
$\nu_{C\text{-}H\ arom.}$	3069, 3001	3070, 3039	3105, 3034
$\nu_{N\text{-}H,\ N\text{-}H^+}$	2944, 2868	2957, –	2970, –
$\nu_{C\text{-}H\ aliph.}$	–	2906	2922
ν_{asCOO^-}	1645	1640 Δ=311	–
ν_{sCOO^-}	1341	1329	–
$\nu_{C=C,\ C=N}$	1516, 1479, 1424	1530, 1492, 1477, 1426	1541, 1489, 1473, 1415
ν_{Cl^-}	–	604, 528	552, 522

Table S2. ^{1}H, ^{13}C and ^{15}N NMR chemical shifts of ruthenium complexes in DMSO-d_6. The coordination shifts ($\Delta_{coord.}$) are shown in parentheses.

Compound	δH2	δC2	δC9	δC10	δN3
Complex**1**	-	145.53(+1.5)	139.82(+3.64)	163.72(+4.99)	–228.41
bimCOOH	-	144.03	136.18	158.73	-
Complex **2**	8.38 (+0.16)	146.32(+4.84)	140.39(+2.99)	-	−219.76
bim	8.22	141.48	137.40	-	–182.97

$\Delta_{coord} = \delta_{complex} – \delta_{ligand}$

Table S3. Crystallographic data and structure refinement details for the arene Ru(II) complexes.

	Complex **1**	Complex **2**
Empirical formula	$C_{18}H_{19}ClN_2O_2Ru$	$C_{17}H_{20}Cl_2N_2Ru$
Formula weight (g/mol)	431.87	424.32
Temperature (K)	120	120
Wavelength of MoKα radiation (Å)	0.71073	0.71073
Crystal system, space group	Monoclinic, $P2_1/n$	Orthorhombic, $Pna2_1$
Unit cell dimensions		
a (Å)	6.5878(3)	6.7975(1)
b (Å)	14.2593(6)	18.2003(3)
c (Å)	18.5967(7)	13.6327(2)
β (°)	94.009(3)	
Volume ($Å^3$)	1742.65(1)	1686.59(4)
Z, density (calculated) (Mg/m^3)	4; 1.646	4; 1.671
Absorption coefficient (mm^{-1})	1.065	1.243
F (000)	872	856
Crystal size (mm)	0.28 × 0.09 × 0.05	0.19 × 0.15 × 0.10
Theta range for data collection θ (°)	2.620 – 29.181	2.988 – 25.494
Index ranges	$-9 \leq h \leq 8, -19 \leq k \leq 19, -25 \leq l \leq 23$	$-8 \leq h \leq 8, -22 \leq k \leq 22, -16 \leq l \leq 16$
Reflections collected/observed [I> 2sigma(I)]	16273/4682 [R_{int} = 0.0245]	35540/3143 [R_{int} = 0.0308]
Completeness to 2θ (%)	2θ = 52.00°; 99.8	2θ = 50.484°; 99.9
Refinement method	Full-matrix least-squares on F^2	
Data/restraints/parameters	4682/0/220	3143/1/202
Goodness-of-fit on F^2	0.933	1.060
Final R indices [I>2sigma(I)]	R_1 = 0.0222, wR_2 = 0.0791	R_1 = 0.0132, wR_2 = 0.0335
R indices (all data)	R_1 = 0.0314, wR_2 = 0.1058	R_1 = 0.0134, wR_2 = 0.0335
Largest diff. peak and hole ($e·Å^{-3}$)	1.197 and –1.555	0.362 and –0.159

Table S4. Percentage of early and late apoptotic and necrotic A549 and VH-10 cells following treatment with ruthenium complexes measured by flow cytometry; mean of three independent experiments ± SD. IP – propidium iodide.

Substance [μM]	A549				VH-10			
	Normal cells (Annexin-/IP-)	Apoptosis Early (Annexin+/IP-)	Apoptosis Late (Annexin+/IP+)	Necrosis (Annexin-/IP+)	Normal cells (Annexin-/IP-)	Apoptosis Early (Annexin+/IP-)	Apoptosis Late (Annexin+/IP+)	Necrosis (Annexin-/IP+)
Control	96.55 ± 0.71	0.30 ± 0.00	3.00 ± 0.56	0.10 ± 0.00	93.00 ± 2.85	0.13 ± 0.05	6.00 ± 2.91	0.90 ± 0.26
$NiCl_2$ positive control	5.90 ± 0.14	0.90 ± 0.07	88.60 ± 0.42	4.70 ± 0.07	48.80 ± 2.85	0.30 ± 0.05	49.70 ± 2.91	1.20 ± 0.26
30 Complex **1**	97.15 ± 0.21	0.35 ± 0.07	2.10 ± 0.14	0.40 ± 0.14	94.85 ± 0.07	0.10 ± 0.00	4.10 ± 0.42	0.95 ± 0.49
30 Complex **2**	98.10 ± 0.14	0.20 ± 0.00	1.55 ± 0.21	0.15 ± 0.07	92.90 ± 0.14	0.30 ± 0.14	6.15 ± 0.07	0.60 ± 0.14
30 bimCOOH	96.70 ± 0.14	0.45 ± 0.07	2.60 ± 0.42	0.20 ± 0.14	88.95 ± 0.49	0.30 ± 0.00	10.00 ± 0.84	0.80 ± 0.28
60 Complex **1**	96.75 ± 0.49	0.30 ± 0.00	2.55 ± 0.49	0.35 ± 0.07	95.85 ± 0.49	0.15 ± 0.07	3.20 ± 0.42	0.85 ± 0.07
60 Complex **2**	98.20 ± 0.00	0.25 ± 0.07	1.45 ± 0.07	0.10 ± 0.00	93.45 ± 0.21	0.40 ± 0.00	5.50 ± 0.42	0.70 ± 0.14
60 bimCOOH	96.65 ± 0.21	0.40 ± 0.00	2.75 ± 0.35	0.20 ± 0.14	88.00 ± 1.97	0.45 ± 0.07	10.40 ± 2.40	1.20 ± 0.42
125 Complex **1**	96.40 ± 0.56	0.30 ± 0.00	2.90 ± 0.70	0.40 ± 0.14	94.90 ± 1.27	0.15 ± 0.07	4.15 ± 1.06	0.85 ± 0.07
125 Complex **2**	97.75 ± 0.07	0.30 ± 0.00	1.90 ± 0.14	0.15 ± 0.07	89.40 ± 0.98	0.55 ± 0.07	9.30 ± 1.41	0.70 ± 0.42
125 bimCOOH	95.55 ± 0.21	0.65 ± 0.07	3.70 ± 0.28	0.15 ± 0.07	79.75 ± 1.48	0.05 ± 0.07	10.90 ± 1.13	9.30 ± 2.68
250 Complex **1**	96.25 ± 0.49	0.35 ± 0.07	3.05 ± 0.49	0.30 ± 0.14	95.40 ± 0.42	0.15 ± 0.07	3.65 ± 0.49	0.90 ± 0.14
250 Complex **2**	97.55 ± 0.21	0.20 ± 0.00	2.05 ± 0.21	0.20 ± 0.00	95.25 ± 0.35	0.20 ± 0.00	4.05 ± 0.49	0.50 ± 0.14
250 bimCOOH	96.30 ± 0.56	0.40 ± 0.00	3.15 ± 0.63	0.15 ± 0.07	81.80 ± 0.28	0.00 ± 0.00	8.20 ± 0.14	10.00 ± 0.42
500 Complex **1**	96.25 ± 0.21	0.30 ± 0.00	3.20 ± 0.14	0.20 ± 0.14	95.50 ± 1.13	0.10 ± 0.00	3.50 ± 0.56	0.95 ± 0.49
500 Complex **2**	97.25 ± 0.07	0.25 ± 0.07	2.45 ± 0.21	0.10 ± 0.00	93.95 ± 0.49	0.35 ± 0.07	4.95 ± 0.63	0.70 ± 0.14
500 bimCOOH	95,70 ± 0.28	0.55 ± 0.07	3.50 ± 0.42	0.30 ± 0.14	79.90 ± 0.14	0.00 ± 0.99	8.00 ± 0.70	12.05 ± 0.63
1000 Complex **1**	93.45 ± 1.48	0.40 ± 0.14	5.70 ± 1.41	0.45 ± 0.07	94.55 ± 1.90	0.10 ± 0.00	4.30 ± 1.83	1.00 ± 0.00
1000 Complex **2**	93.25 ± 0.49	0.45 ± 0.07	6.00 ± 0.07	0.35 ± 0.21	91.50 ± 0.76	0.20 ± 0.30	6.30 ± 0,07	2.00 ± 0.00
1000 bimCOOH	91.95 ± 0.35	0.85 ± 0.07	6.95 ± 0.35	0.25 ± 0.07	70.50 ± 2.12	0.00 ± 0.00	14.00 ± 1.13	15.50 ± 3.25

2. Supporting figures

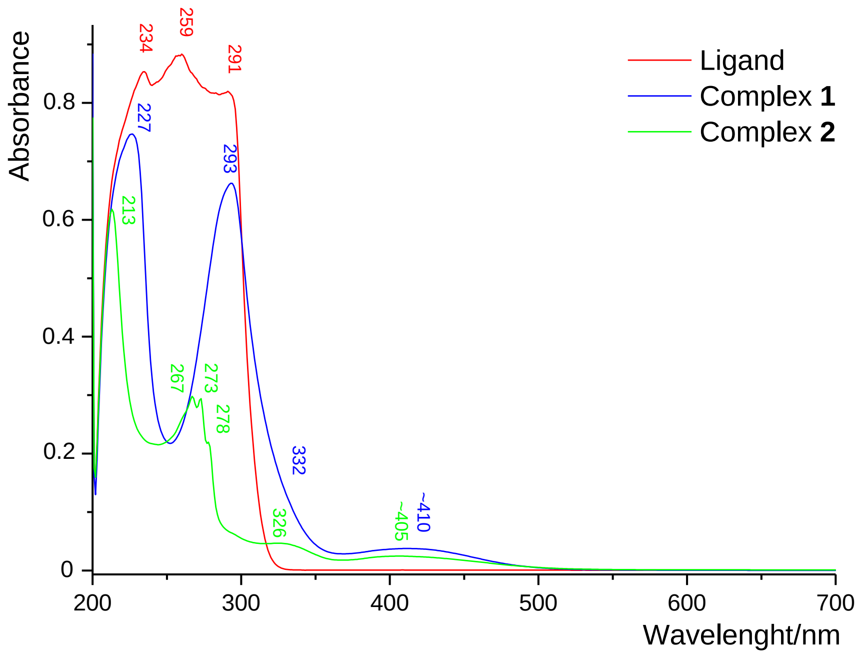

Figure S1. UV-vis spectra of 1*H*-benzimidazole-2-carboxylic acid, complex **1** and complex **2** in methanol at 298 K.

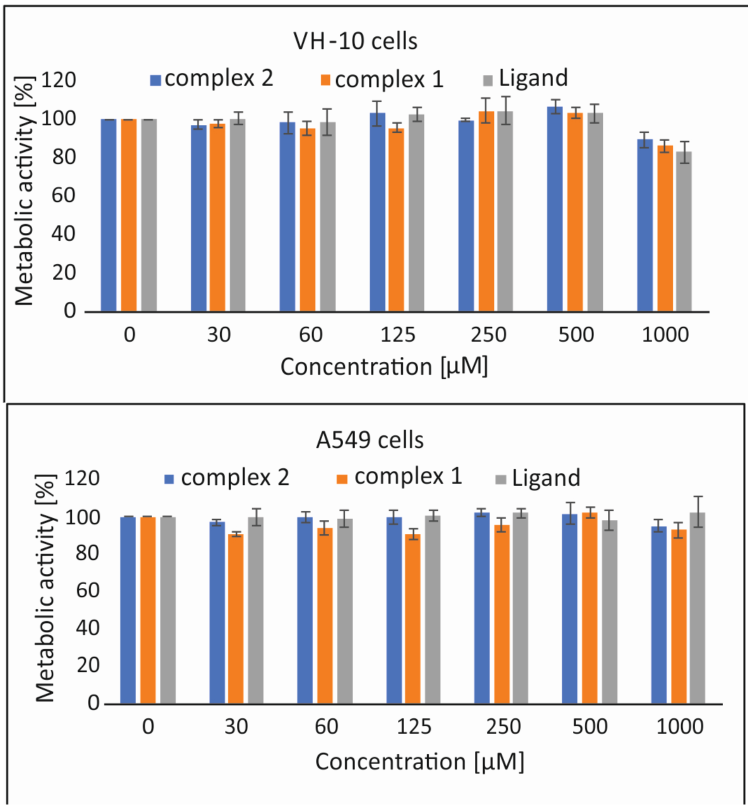

Figure S2. The MTS percentage of VH-10 and A549 cells metabolic activity under an increasing dose of ruthenium complexes or their ligand alone after 24 hours treatment.

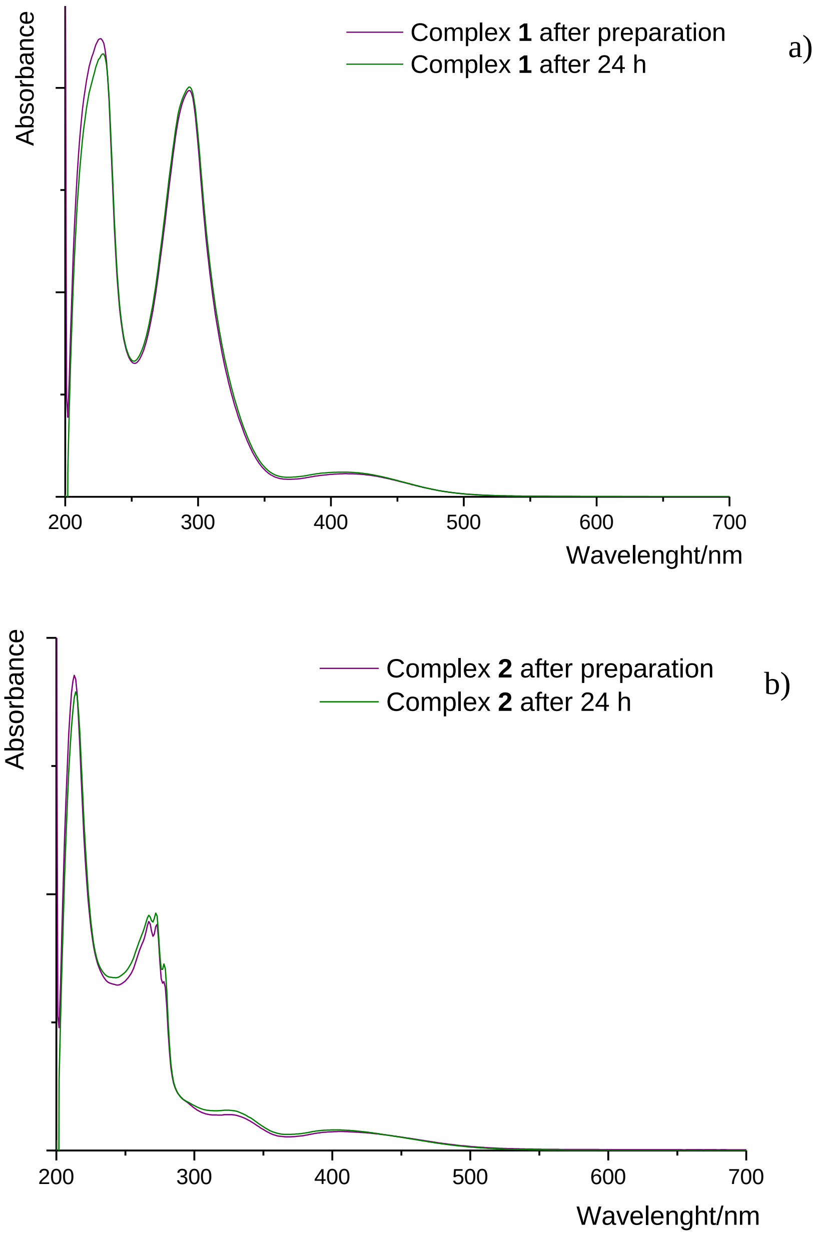

Figure S3. UV-vis spectra of the complexes **1** (a) and **2** (b) in methanol solution, after preparation and after 24 h.

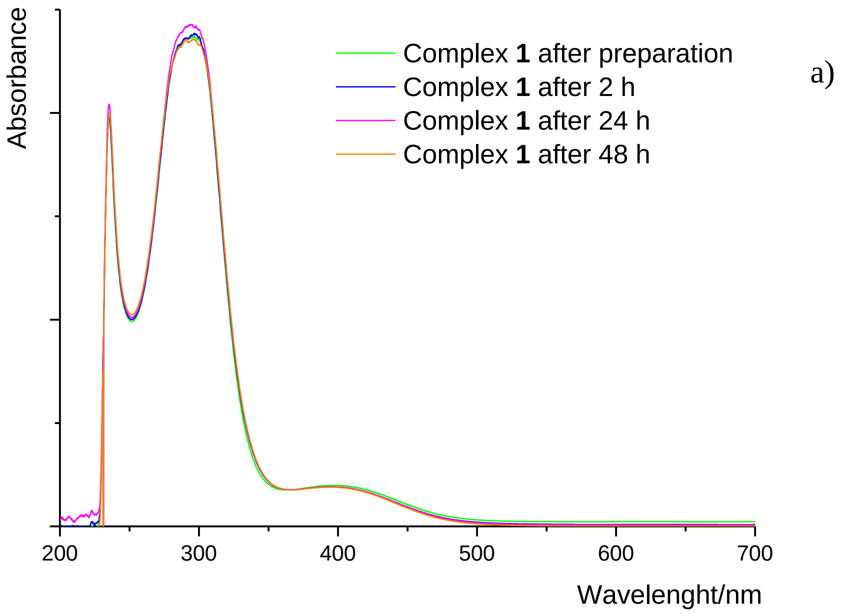

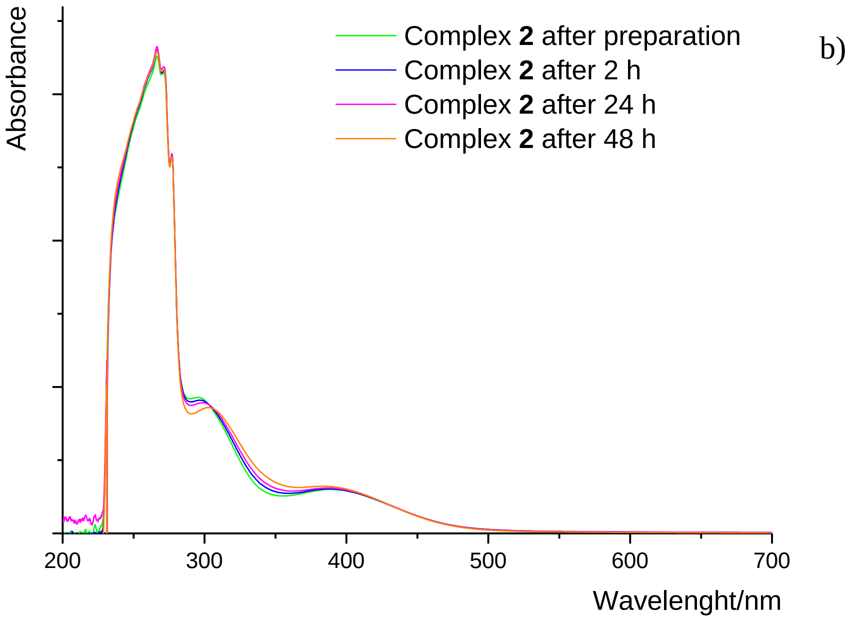

Figure S4. Time dependence of UV-vis spectra of the complexes **1** (a) and **2** (b) in H_2O/DMSO mixture.
